# Rapid mapping and cloning of the virescent-1 gene in cotton by bulked segregant analysis–next generation sequencing and virus-induced gene silencing strategies

**DOI:** 10.1093/jxb/erx240

**Published:** 2017-07-20

**Authors:** Jiankun Zhu, Jiedan Chen, Fengkai Gao, Chenyu Xu, Huaitong Wu, Kun Chen, Zhanfeng Si, Hu Yan, Tianzhen Zhang

**Affiliations:** 1 State Key Laboratory of Crop Genetics and Germplasm Enhancement, Cotton Hybrid R & D Engineering Center (the Ministry of Education), Nanjing Agricultural University, Nanjing, China; 2 Agronomy Department, College of Agriculture and Biotechnology, Zhejiang University, Zhejiang, China

**Keywords:** Bulked segregant analysis, cotton, map-based clone, VIGS, virescent

## Abstract

Map-based gene cloning is a vital strategy for the identification of the quantitative trait loci or genes underlying important agronomic traits. The conventional map-based cloning method is powerful but generally time-consuming and labor-intensive. In this context, we introduce an improved bulked segregant analysis method in combination with a virus-induced gene silencing (VIGS) strategy for rapid and reliable gene mapping, identification and functional verification. This method was applied to a multiple recessive marker line of upland cotton, Texas 582 (T582), and identified unique genomic positions harboring mutant loci, showing the reliability and efficacy of this method. The *v*_*1*_ locus was further fine-mapped. Only one gene, *GhCHLI*, which encodes one of the subunits of Mg chelatase, was differentially down-regulated in T582 compared with TM-1. A point mutation occurred in the AAA+ conserved region of *GhCHLI* and led to an amino acid substitution. Suppression of its expression by VIGS in TM-1 resulted in a yellow blade phenotype that was similar to T582. This integrated approach provides a paradigm for the rapid mapping and identification of the candidate genes underlying the genetic traits in plants with large and complex genomes in the future.

## Teaser

An improved method of rapid gene mapping and identification was successfully used to map and identify the causal gene at the virescent-1 locus of upland cotton.

## Introduction

Map-based cloning is an important strategy for the identification of candidate genes underlying particular mutant phenotypes. Map-based cloning is a process based on the gene location on the chromosome, so it is not necessary to make prior assumptions or to have knowledge of the specific genes. Since 1992, when this approach was initially applied to the identification of *FAD3* and *ABI3* in Arabidopsis (Arondel *et al.*, 1992; Giraudat *et al.*, 1992), more than 130 genes or quantitative trait loci (QTLs) have been cloned, such as the Arabidopsis gene *AXR1* (Leyser *et al.*, 1993), the rice bacterial leaf blight resistance genes, *Xa21* (Song *et al.*, 1995) and *Xa1* (Yoshimura *et al.*, 1998), the rice blast resistance gene, *Pi-b* (Rybka *et al.*, 1997), the control of rice tillering gene, *Moc1* (Li *et al.*, 2003), the wheat nematode resistance gene, *Cre3* (de Majnik *et al.*, 2003), the beet Hs1pro cyst nematode resistance gene 1 (Thurau *et al.*, 2003) and the tomato vegetable fruit weight QTLs, *FW2.2* (Frary *et al.*, 2000) and *FW3.2* (Chakrabarti *et al.*, 2013). However, the development and screening of the DNA markers for the purpose of linkage analysis in map-based cloning constitutes time-consuming and cumbersome work. Bulked segregant analysis (BSA) constitutes an elegant method for rapid screening markers such as RAPDs and RFLPs to detect the most closely linked markers (Giovannoni *et al.*, 1991; Michelmore *et al.*, 1991). Two bulked pools composed of individuals with extreme traits were constructed, and were then screened with arbitrary primers until the markers that co-separated with the bulked pool of the extreme phenotype were screened out. Such a strategy, which can be used to exclude the unlinked markers in a rapid manner, and to identify the genomic region of the target loci, has already been successfully applied to the screening out of markers linked to genes/QTLs for important agronomic traits in many crops, such as soybean (Vuong *et al.*, 2016), wheat (Lu *et al.*, 2015) and rice (Xu *et al.*, 2011; Fang *et al.*, 2015). Coupled with the traditional map-based cloning procedure, namely BSA with whole genome sequencing, some improved methods for the mapping and isolation of genes at specific genetic loci have been developed, such as MutMap, QTL-seq, and Mutmap+ (Abe *et al.*, 2012; Fekih *et al.*, 2013; Takagi *et al.*, 2013). These approaches have been proven to be powerful, efficient and cost-effective. For instance, MutMap was employed in the discovery of the gene conferring the salinity-tolerance phenotype of *hst1*, which contributed to the breeding of a salt-tolerant rice cultivar in only 2 years (Abe *et al.*, 2012; Takagi *et al.*, 2015). However, progress in gene isolation using map-based cloning in non-model plants with complex and large genomes, such as cotton and wheat, lagged behind that of other crops. Cotton is a tetraploid species with a genome size of 2.5 Gb and a high content of repetitive elements (Zhang *et al.*, 2015*b*). Due to its genome complexity, for a long period almost no genes were identified using the map-based cloning strategy, although many genetic loci or QTLs controlling the favorable traits have been mapped with closely linked DNA markers in the cotton genome. Recently, the release of the draft cotton genome (Paterson *et al.*, 2012; Li *et al.*, 2014; Liu *et al.*, 2015; Zhang *et al.*, 2015*b*) has greatly promoted map-based gene cloning, but only a small number of pivotal genes responsible for important agricultural traits in cotton have been identified and functionally validated, such as the dominant glandless gene, *Gl*_*2*_^*e*^ (Ma *et al.*, 2016), dominant fibreless gene, *N*_*1*_ (Wan *et al.*, 2016), and Okra leaf gene, *L*_*2*_ (Chang *et al.*, 2016*b*).

Since the 1930s, 198 genetic loci for different qualitative characters have been identified in cotton (Pan, 1998). These morphological mutants are valuable sources for genetic research and some loci have agronomic value in cotton breeding and production. Killough and Horlacher (1933) identified the first virescent mutant, and assigned the gene symbol *v*_*1*_. The seedlings and young plants of *v*_*1*_ mutant cotton had a greenish yellow appearance, but as the plant developed, the leaves adopted a normal green appearance. Inheritance tests have shown that the *v*_*1*_ character is determined by one pair of recessive genes. Simple inherited virescent yellow is easily distinguishable in the seedling stage and can be used to develop cultivars with indicative characters, and parents for use in the production of hybrid seeds. Upon transferring the virescent gene into the commercial cultivars by means of backcrossing, Hua *et al.* (1963) developed parental virescents in order to produce interspecific hybrids between *Gossypium hirsutum* and *G. barbadense* in China. Duncan and Pate (1967) and our laboratory conducted a vast amount of research into hybrid seed production using indicative virescent characteristics. The homozygous virescent plants could be easily identified and eliminated during the ordinary practices of thinning and weeding from the F_1_ hybrids. It was proven that this is a feasible procedure (Wang and Pan, 1989; Pan *et al.*, 1993; Min *et al.*, 1996).

Texas 582 (T582) is a multiple recessive marker line with the same genetic background as TM-1 and the genetic standard line of the upland cotton (Kohel *et al.*, 1965). It contains five mutant phenotypes controlled by five recessive alleles at a single locus, namely the virescent-1 (*v*_*1*_), cup leaf (*cu*), glandless-1 (*gl*_*1*_), frego bract (*fg*) and cluster-1 (*cl*_*1*_) alleles. In this report, we integrated traditional map-based cloning, BSA and next generation sequencing (designated as BSA-seq), and virus-induced gene silencing (VIGS) strategies for the rapid mapping and identification of a causal gene for *v*_*1*_. This work provides a paradigm for the rapid map-based gene cloning of plants with large and polyploid genomes.

## Materials and methods

### Plant material

For the purpose of genetic analysis, an F_2_ population and a BC_1_ population were developed. T582 plants were crossed with TM-1 plants, and the F_1_ plants were self-pollinated to produce an F_2_ population that consisted of 2164 individuals at the Jiangpu Breeding Station, Nanjing Agriculture University. The F_1_ plants were also crossed with T582 to produce a BC_1_ population consisting of 412 individuals. These experiments were conducted in the Hainan Breeding Station of the Nanjing Agriculture University in the winter of 2014. A total of 483 of the 2164 F_2_ plants were used to map the mutant loci through SSR markers, and the BC_1_ population cultivated at Jiangpu Breeding Station in the summer of 2015 was used to select individual mutant traits in order to construct sequencing pools. The phenotypes of the F_1_, F_2_ and BC_1_ populations were investigated. The F_2_ and BC_1_ segregation ratios were analyzed with a χ^*2*^ goodness of fit test using Microsoft Excel software.

### Cotton genome DNA extraction and sequencing pool construction

Since T582 contains five different mutant traits, and each BC_1_ individual includes at least one mutant trait, we surveyed the yellowish leaf characteristic for *v*_*1*_, the upper curved leaf character for *cu*, stem and bolls without glands for *gl*_*1*_, the narrow bract characteristic for *fg* and the twin bolls characteristic for *cl*_*1*_, and it was observed that each individual contained one or more extreme characteristics. We furthermore sampled 28 recessive extreme individuals from 412 progenies of BC_1_. Then, we extracted genomic DNA from the recessive extreme individuals and the mutant parent T582 by means of the CTAB method (Paterson *et al.*, 1993). The genomic DNA of 28 mutant progenies was bulked in an equal ratio in order to generate a mutant-type bulked DNA pool. All five bulked DNA pools and T582 genomic DNA were subjected to sequencing on the Illumina HiSeq 2500 platform using the 2 × 101 bp model.

### Alignment of short reads to the TM-1 reference

The Illumina reads were firstly trimmed with Sickle (https://github.com/najoshi/sickle) using a PHRED quality threshold of 20 and a minimum length threshold of 70. After the low-quality reads were filtered out, the remaining reads were aligned to the *Gossypium hirsutum* TM-1 reference genome using BWA software (Li *et al.*, 2009*a*). SAMtools (Li *et al.*, 2009*b*) was used to convert the mapping results to a BAM format, and duplicated reads were filtered out using the Picard package (http://broadinstitute.github.io/picard).

### Identification of SNP and InDel between T582 and TM-1

Homozygous single nucleotide polymorphisms (SNPs) were first called by alignment of reads from the parental line, T582, to the reference genome sequences of TM-1 using SAMtools and an in-house Perl script under the following conditions: the minimum read depth was 10 and the allele represented at least 90% of all the alleles observed. InDels were identified by SAMtools software (samtools mpileup – C50 – Q20 – q20) and InDel primer pairs were designed based on the flanking sequences of identified InDels using PRIMER3 software (Untergasser *et al.*, 2012).

### BSA-seq analysis

Based on the two parental SNP datasets, the BAM format files of the BC_1_ bulked samples were converted to the plain-text format ‘pileup’ files, encapsulating the read and base quality data over that position, using SAMtools. Then, the number of short reads corresponding to each of the two parental genomes was evaluated based on ‘pileup’ files for each SNP site. In order to improve the accuracy of the identification of candidate regions, consecutive low depth SNPs were classified into a block with a minimum read depth of 20. The allele frequencies and the statistical significance of the differences in allele frequency (*P* value, Fisher’s exact test) were evaluated in a block. In order to avoid a significant difference in terms of the sequencing error, the alignment error and the error probability of alpha for one test, a sliding window analysis was applied to identify candidate regions with a 2 Mb window size and a 100 kb increment. In the window, the average –log10(*P*) was calculated as follows:

$$\text{average }-\text{log}10\left(P\right)=\frac{\sum_{i}^{n}-log10(P_{i})}{n}$$

where *n* is number of blocks that was used for the Fisher’s exact test in the window. In order to reduce the noise, we filtered out windows with less than five blocks (*n*<5) for the analysis. Genomic regions with an average –log10(*P*) value>2 were identified and considered as candidate regions.

### Measurement of chlorophyll

The leaves second from the top of the 4-week-old cotton plants were picked and cut into 4mm×5mm strips. For chlorophyll extraction, 0.10 g leaves were put into 50-ml tubes with a 25 ml ethanol–acetone mixture (volume ratio 1:1) in the dark for no more than 12 h until the leaves turned white. The extract was measured at absorbance values of 470, 645 and 663 nm by UV spectrophotometer, and the concentrations of chlorophyll *a* and chlorophyll *b* were measured using Arnon’s method (Arnon, 1949).

### RNA extraction and quantitative RT-PCR

Total RNA was extracted from the leaves second from the top of the mutant and normal seedling plants using the Plant RNA Rapid Extraction Kit (Molfarming, China). The total RNA was reversed to cDNA using the HiScript II Reverse Transcriptase Kit (Vazyme, China). The cDNA was diluted to 100 ng µl^−1^ and mixed with SYBR Premix Ex Taq II Kit (Takara, Japan), then used for quantitative PCR (qPCR) with ABI7500 according to the protocol provided by the manufacturer. Primers (see Supplementary Table S1 at *JXB* online) were used to detect the expression of the candidate genes, which produced 70–200 bp fragments. The cotton histone gene was used as an internal reference. The gene expression level was calculated using the ${\text{2}}_{}^{-\Delta \Delta C_{\text{T}}}$ method (Livak and Schmittgen, 2001).

### Transmission electron microscopy analysis

Leaves from TM-1 and T582 plants were observed using a transmission electron microscope. Transverse sections of the leaf samples were fixed in 2.5% glutaraldehyde in a phosphate buffer and further fixed overnight in 1% OsO4 at 4 °C. After staining with uranyl acetate, the tissues were further dehydrated through the application of ethanol, and then embedded in Spurr’s medium prior to ultrathin sectioning. The sections were air dried, stained again, and viewed with a Hitachi H-7650 transmission electron microscope (Zhou *et al.*, 2017). In order to count the number of chloroplasts and to survey the structure of the chloroplasts, 20 cells were examined from each sample.

### Virus-induced gene silencing (VIGS) assays

In order to knockdown the expression of the *GhChlI* gene, a 337-bp fragment of the *GhChlI_D10* cDNA from TM-1 was PCR-amplified using Pfu DNA polymerase (Vazyme) and primers K7002F and K7002R (see Supplementary Table S1). The resulting PCR product was recombined into *Eco*RI–*Bam*HI-cut pTRV2 in order to produce a VIGS vector named pTRV2-*ChlI_D10*. The pTRV1 and pTRV2-*ChlI_D10* vectors were introduced into the Agrobacterium strain GV3101 by means of electroporation (Bio-Rad, Hercules, CA, USA). For the VIGS assay, the transformed Agrobacterium colonies containing pTRV1 and pTRV2-*ChlI_D10* were grown overnight at 28 °C in an antibiotic medium containing rifampicin and kanamycin in proportions of 50 mg l^−1^ each. The Agrobacterium cells were collected and resuspended in the infiltration medium (10 mM MgCl_2_, 10 mM MES and 200 mM acetosyringone) and subsequently adjusted to an OD_600_ of 0.5. The Agrobacterium strains containing the TRV1 and TRV2 vectors were mixed at a ratio of 1:1. Seedlings with mature cotyledons but without a visible rosette leaf (7 days after germination) were infiltrated by inserting the Agrobacterium suspension into the cotyledons via a syringe. The plants were grown at 23 °C in pots arranged in a growth chamber under a 16-h light–8-h dark cycle and at a humidity rate of 60% (Ma *et al.*, 2016). Gao *et al.* (2011*a*,b) reported the protocol of Agrobacterium-mediated VIGS system in cotton, and took the *cloroplastos alterados 1* gene (*GrCLA1*) as an example to prove the system was working. *CLA1* encodes a 1-deoxyxylulose 5-phosphate synthase that plays an important role in chloroplast development in plants and is highly conserved. The mutant *cla1-1* has an obvious albino phenotype in the entire plant during seeding stage (Mandel *et al.*, 1996), and Gao *et al* showed that loss-of-function of *GrCLA1* resulted in an albino phenotype on true leaves. The sequence of *GhCLA1* is highly similar to *GrCLA1*, and therefore we used *GhCLA1* as the positive control for VIGS.

### Gene cloning and multiple sequence alignment

Genomic DNAs of the eight open reading frames (ORFs) were amplified from both TM-1 and T582 using gene-specific primers (see Supplementary Table S1). The sequence of ORF1, ORF2, ORF3, ORF4, ORF6 and ORF7 were amplified by ExTaq Kit (Takara, Japan) and cloned into the pMD-19T Vector (Takara, Japan) for sequencing. The sequence of ORF5, ORF8 and promoter of ORF4 were amplified by Phanta Super-Fidelity DNA Polymerase Kit (Vazyme), and then the amplified product was linked to the CE Entry Vector (Vazyme) using ClonExpress Entry One Step Cloning Kit (Vazyme). The resulting plasmid was transformed into DH5α competent cells for sequencing. Multiple sequence alignment was performed with ClustalX software using multiple alignment modes.

## Results

### Rapid mapping of the mutant genetic loci by BSA-seq

**Fig. 1.**
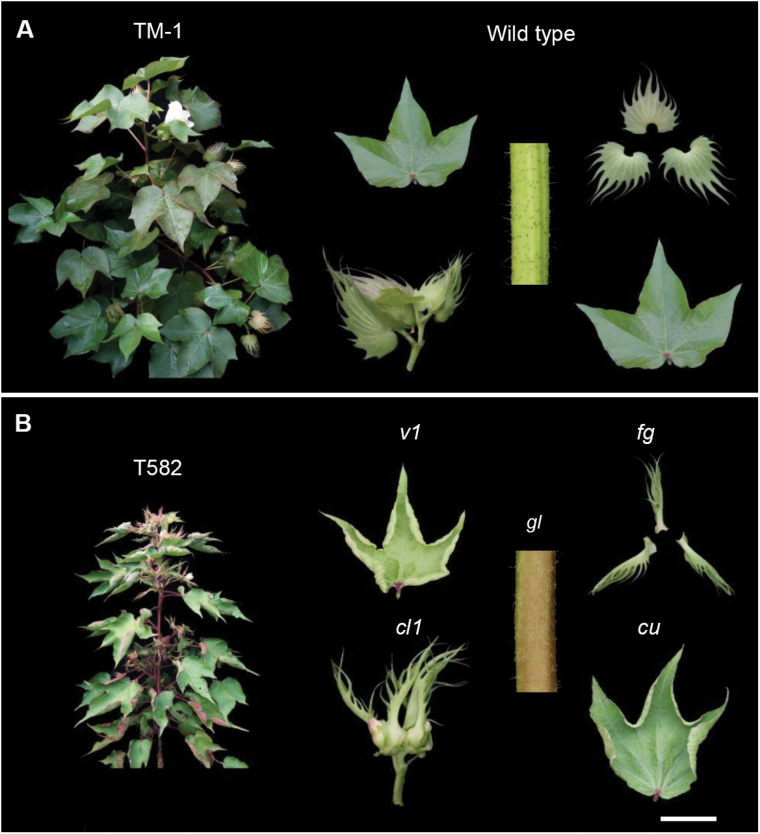
Characterization of the T582 phenotypes. (A) Five TM-1 phenotypes. (B) T582 includes five recessive traits, namely virescent-1 (*v*_*1*_), cup leaf (*cu*), glandless-1 (*gl*_*1*_), frego bract (*fg*) and cluster-1 (*cl*_*1*_). Scale bar: 2 cm.

**Fig. 2.**
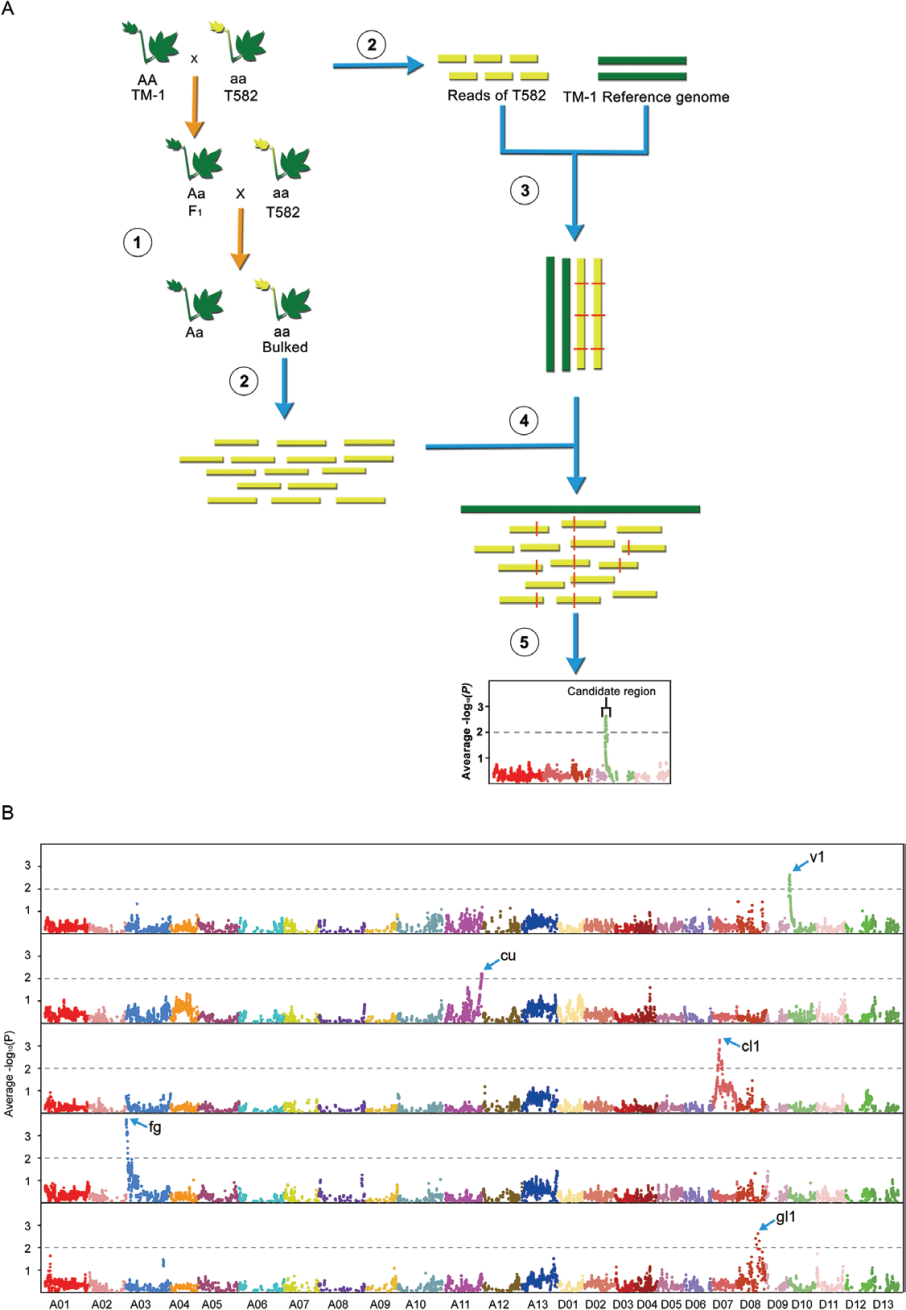
Simplified scheme of the application of BSA-seq. (A) Step1: the *v*_*1*_ mutant was crossed with the wild-type to generate the BC_1_ progeny, which were segregated according to the mutant and wild-type phenotypes. Step 2: the DNA from T582 plants harboring the *v*_*1*_ locus, and the 28 BC_1_ individuals displaying the mutant phenotype were bulked and subjected to whole genome sequencing. Step 3: the T582 reads were aligned to the reference genome TM-1 to produce a set of SNP data between the two parents. Step 4: the reads from the bulked mutant-type pool were mapped to the reference genomes TM-1 and the ratio of SNP distribution calculated in the 28 bulked mutant-type individuals. Step 5: the average –log10(*P*) value was scanned across 26 cotton chromosomes to detect the genomic regions composed only of mutant-type SNPs. SNPs with sequence reads composed only of mutant sequences are closely linked to the causal SNP for the mutant phenotype. (B) The genomic intervals of the five genetic loci, as identified by BSA-seq analysis. At the level of –log10(*P*)>2, distinct peaks, marked by blue arrows, served as the regions spanning the candidate genes. The *v*_*1*_ locus was mapped to chr. D10, *cu* to chr. A11, *cl*_*1*_ to chr. D07, *fg* to chr. A03, and *gl1* to chr. D08.

For the rapid mapping and identification of the genomic regions that contribute to the traits of interest (*v*_*1*_, *cu*, *gl*_1_, *fg*, *cl*_*1*_) in T582 (Fig. 1), we performed the improved BSA-seq method, which combined the traditional map-based cloning strategy with BSA cloning based on next generation sequencing (Fig. 2A). Initially, we generated a backcross population by crossing T582 and TM-1. Then, we extracted DNA from 28 individuals from the BC_1_ progenies corresponding to the five mutant phenotypes. The prepared DNA was bulked in an equal ratio in order to generate the ‘mutant type’ pools. Thus, five mutant bulks were generated for the five mutant phenotypes. All pools, as well as the DNA extracted from the parent T582, were subjected to whole-genome resequencing. A total of 221.6 Gb of short (101-bp) paired-end reads was identified, including 94.7 Gb reads (37.9-fold genome coverage) for T582 and an average of 25.4 Gb ranging from ~17.9 to ~34.4 Gb (7.2~13.8-fold genome coverage) for the five bulks for the different mutant traits. These reads were trimmed using Sickle software and then aligned to the TM-1 reference genome (Zhang *et al.*, 2015*b*). A total of 347 629 SNPs were identified between the two parents, TM-1 and T582. Then, the reads from the mutant bulked pools were aligned respectively in order to calculate the ratio of the number of reads corresponding to the two parental genomes. In principle, the causative genomic regions should be shared by all T582-type BC_1_ plants, whereas the genomic regions unrelated to the T582 phenotype should segregate randomly among the BC_1_ progenies with mutant traits. In order to identify the genomic regions related to the T582 genotype, the allele frequencies and the statistical significance of the difference in allele frequency (*P* value, Fisher’s exact test) were evaluated in a block with a minimum reading depth of 20 of consecutive SNPs across the whole genome. In order to avoid a significant difference in terms of the sequencing and the alignment error, an average –log_10_(*P*) value was applied. Genomic regions with an average –log_10_(*P*) value>2 were identified and considered as the candidate regions. In this manner, five genetic loci were anchored to specific regions on the chromosomes. As a result, the *v*_*1*_ locus was anchored to the interval between 0.7 Mb and 3.9 Mb on chromosome D10 (chr.20) according to the TM-1 reference genome (Zhang *et al.*, 2015*b*) (Fig. 2B, Supplementary Table S2). We also mapped *cu* to chr. A11, *gl*_*1*_ to chr. D08, *fg* to chr. A03, and *cl*_*1*_ to chr. D07 (Fig. 2B, Supplementary Table S2). In order to verify the reliability of the causal gene identification for a given phenotype by the BSA-seq approach, the *v*_*1*_ locus was taken as a guide for further tests. Due to their linkage relationship, genomic sequences in the proximity of the mutant site would be passed on to the progenies, and therefore BC_1_ individuals displaying the mutant phenotype should share a common consecutive sequence derived from T582. Based on this, we checked the sequence origin of the candidate regions and detected several sequences in the candidate regions that were identical to those in T582, representing the most probable regions for the mutant *v*_*1*_ loci. Consequently, the *v*_*1*_ locus was narrowed down to two small intervals (2.42–2.7 Mb, 0.29 Mb in length, 3.37–2.87 Mb, 0.51 Mb in length) (see Supplementary Table S2).

### Validation of the BSA-seq mapping result

To further confirm the mapping accuracy of this approach, a genetic linkage analysis was carried out to characterize the linkage relationship between the mutant loci and the molecular markers. We developed an additional F_2_ population by crossing TM-1 and T582. This F_2_ contained a total of 2164 individuals. A 3:1 ratio of wild-type to mutant plants was scored in this progeny, confirming that the mutant trait is conferred by a single recessive mutation gene (Table 1). Of the 3324 molecular markers developed in our lab (Zhao *et al.*, 2012), 274 polymorphic markers were screened out between TM-1 and T582. Using these polymorphic primers to screen 438 individuals of the F_2_ population, the NAU2540 marker was found to be tightly linked to *v*_*1*_. The sequences of these primers were located on the corresponding genomic segments identified by the BSA-seq. The linkage analysis therefore demonstrates that the BSA-seq approach is efficient and accurate for identifying target loci in cotton.

**Table 1.** *Segregation ratio for the* v_1_*mutant and wild-type categories in the mapping populations*

Cross/generation	Total plants	Green plants	Yellow plants	Expected ratio	χ^2 *a*^
(T582×TM-1)BC_1_	412	218	194	1:1	1.398
(T582×TM-1)F_2_	2164	1661	503	3:1	3.559

^*a*^ χ^2^ test for goodness-of-fit at 0.05 significance level (χ^2^_0.05:1_=3.84).

### Isolation and functional verification of the candidate gene underlying the *v*_*_1_*_ locus

**Fig. 3.**
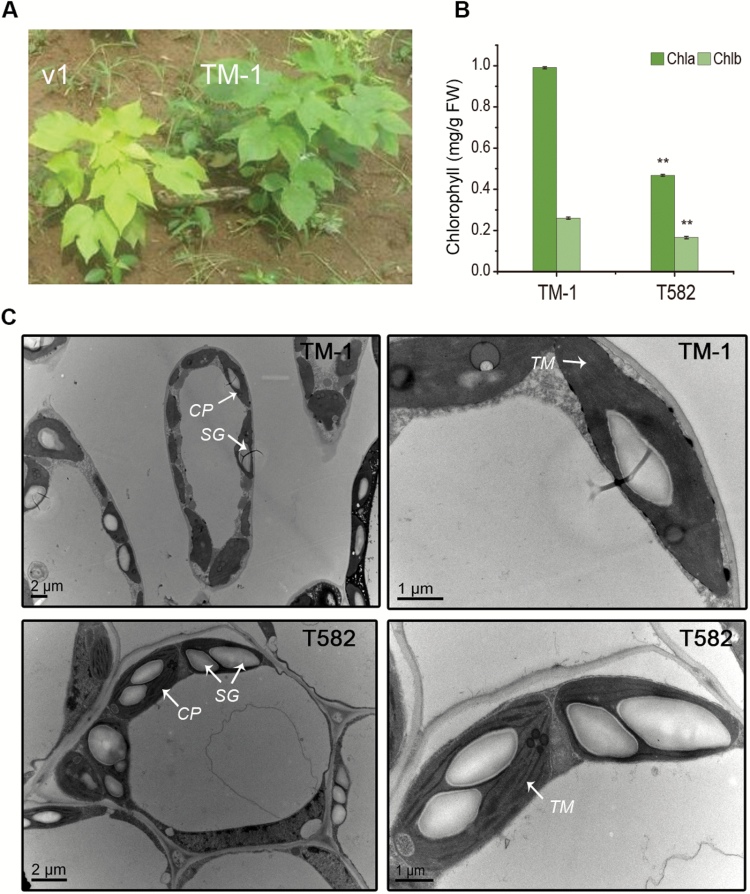
Morphological and biological characteristics of *v*_*1*_ mutant. (A) Wild-type and *v*_*1*_ mutant phenotypes at the seedling stage. (B) Comparison of the chlorophyll *a* (Chla) and chlorophyll *b* (Chlb) content of T582 and TM-1 leaves. The error bars represent the standard deviation of three biological repeats, and ** indicates significant differences compared with the corresponding wild-type TM-1 at *P*<0.01 by Student’s *t* test. (C) Transmission electron microscopy of the chloroplast ultrastructure of TM-1 and T582 leaves. Electron micrographs showing mesophyll cells and chloroplasts of TM-1 (above) and T582 (below). CP, chloroplast; SG, starch grain; TM, thylakoid membrane.

Under field growth conditions, T582 seedling leaves exhibited a canary phenotype (Fig. 3A): the elder leaves turned green but the new leaves were still yellow when the plant had entered the post-flowering period in late summer. The leaf color of the heterozygote F_1_ TM-1 and T582 hybrid plants was green. The proportion of plants with green leaves and yellow leaves was consistent with the segregation ratios of 3:1 and 1:1 in the F_2_ and BC_1_ populations, respectively, indicating that the trait was controlled by a recessive gene (Table 1). The total chlorophyll content of the second from the top leaves was significantly lower in T582, by about 50% compared with the wide-type parent, TM-1 (Fig. 3B). Transmission electron microscopy demonstrated a significantly lower chloroplast number per cell in the T582 mesophyll cells than those in TM-1 (Student’s *t* test, *P*=2.63 × 10^−7^). Most of the chloroplasts in the mutant exhibited fewer lamellar structures than the wild-type, no granum lamella or stroma lamella and the reaction center containing most of the chlorophyll was in the membrane of the thylakoid (Fig. 3C). As such, it is estimated that the *v*_*1*_ gene mutation disrupts the formation of thylakoids in the chloroplast and causes chlorophyll accumulation.

**Fig. 4.**
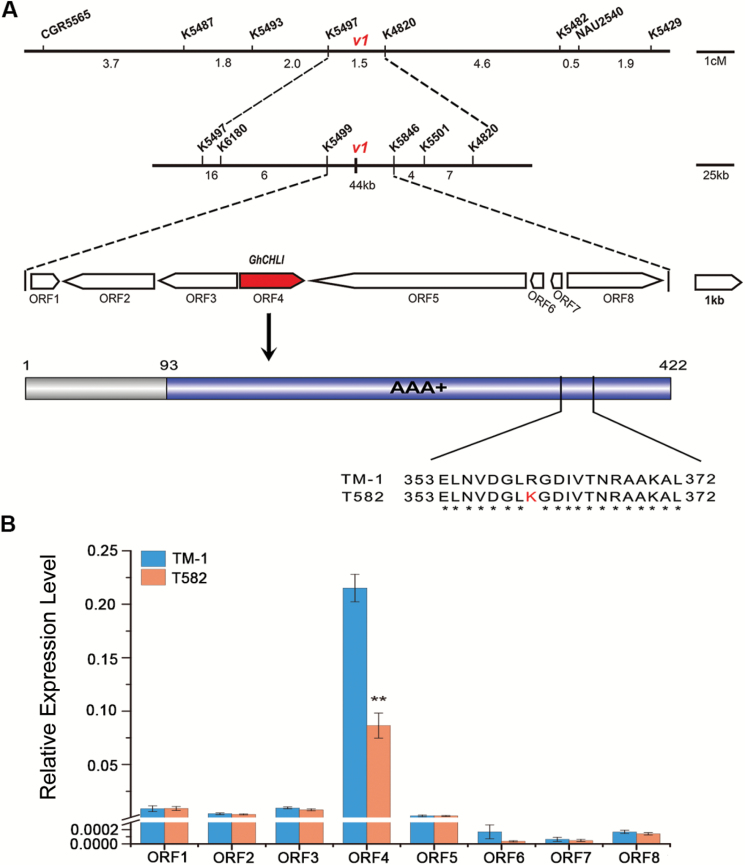
Map-based cloning of the *v*_*1*_ gene. (A) Fine mapping of *v*_*1*_. The *v*_*1*_ locus was narrowed to a 44.21kb interval that included eight ORFs. ORF4 was the candidate gene, and the 1366th base, G, had changed to A in T582, resulting in the 361st amino acid being substituted from arginine (R) to lysine (K) in the AAA+ conserved region of *GhCHLI*. (B) Relative expression of eight candidate ORFs; error bars represent standard deviation (SD) of three biological repeats; ** indicates significant differences compared with the corresponding wild-type TM-1 at *P*<0.01 according to a Student’s *t* test.

To determine more precisely the interval of the *v*_*1*_ locus, 126 541 InDels were identified based on the alignment of T582 Illumina reads to the TM-1 genome reference. On combining these data with the genomic region identified by BSA-seq, 83 of the InDels were selected for screening of the mapping population. As a result, the *v*_*1*_ locus was anchored between InDels K5497 and K4820, with a genetic distance of 1.5 cM (Fig. 4A). The genetic map was constructed based on data from 131 F_2_ individuals and oriented by integrating the reference framework SSRs and InDels using Joinmap 4.1 (Van Ooijen, 2011). Then, to shorten the physical mapping interval, an enlarged mapping population comprising 2622 individuals was further used for fine mapping. Finally, the *v*_*1*_ locus was delimited to a 44-kb region flanked by two newly developed InDel-type markers, K5499 and K5846 (Fig. 4A). Within this region, eight putative ORFs are predicted according to the reference genome sequence of the tetraploid cotton *G. hirsutum acc*. TM-1 (PRJNA248163) (see Supplementary Table S3).

Then, we integrated SNP analysis, DNA sequence alignment, expression profiling in various tissues, the expression level comparison of TM-1 and T582, function annotation of the orthologues in Arabidopsis, and VIGS to rapidly identify the candidate genes responsible for the mutant trait. First, we compared the ORF sequences from TM-1 and T582 and coding DNA sequence from TM-1. The alignment showed no sequence variants in the coding region between TM-1 and T582 of other ORFs except ORF1 and ORF4 (see Supplementary Figs S1–S8). An SNP occurred in one of the exons of ORF1 and ORF4, which resulted in the respective non-synonymous mutations. To examine the linkage relationship of the identified SNP with the *v*_*1*_ locus, 503 virescent F_2_ individuals were subjected to linkage analysis. Results showed that the SNP in ORF1 was not consistent in the mutant F_2_ individuals (Supplementary Fig. S9) while the SNP in the ORF4 was co-segregated with the *v*_*1*_ locus (Supplementary Fig. S10). We further sequenced the ORF4 gene from the 20 virescent individuals and sequence alignments confirmed that they all shared this allelic variation, the same as in T582, further suggesting this SNP was the causative nucleotide variation for the *v*_*1*_ phenotype in T582 (Fig. S11). Furthermore, quantitative RT–PCR (qPCR) showed that among the eight candidate ORFs, ORF4 was significantly down-regulated by at least two-fold in leaves of T582 plants compared with TM-1 plants (Fig. 4B). Taken together, these results confirmed that ORF4 is the most likely candidate gene underlying the *v*_*1*_ locus.

To get more information for ORF4, we isolated the full length and the promoter of the ORF4 gene from TM-1 and T582. The cloned genomic DNA was composed of 1553 bp with a 1269 bp ORF and three exons. The ORF4 gene encoded 422 amino acid residues with a predicted molecular mass of 46 kDa. This gene was annotated as a homologous gene of the Arabidopsis *CHLI* gene, which encodes one of the three subunits of magnesium chelatase I, playing an important role in chloroplast biosynthesis in higher plants. Therefore, we referred to the candidate gene as *GhCHLI*. When compared with TM-1, the single nucleotide transition was present at point 1366 bp (G to A), resulting in substitution of arginine (R) for lysine (K) at the 361st amino acid (Fig. 4, Supplementary Fig. S4). The predicted gene structure indicated that the mutation was located in the highly conserved region (from the 93rd to the 422nd amino acid) that may function as an AAA+ domain necessary for the activity of GhCHLI (Rissler *et al.*, 2002; Ikegami *et al.*, 2007). In addition to sequence variation, the significant lower expression level of *GhCHLI* was simultaneously found in T582. So we isolated the 2-kb fragments upstream of the start codon of *GhCHLI* from TM-1 and T582 to detect promoter variation. But alignment results showed no sequence variation in the promoter sequence between them (Supplementary Fig. S12), indicating that their promoters did not confer the extra low expression of *GhCHLI* in T582.

**Fig. 5.**
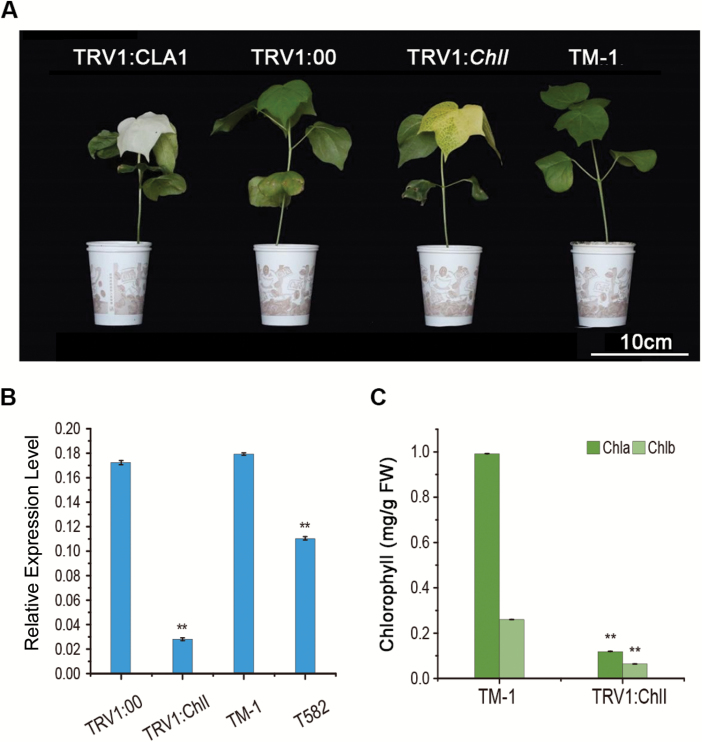
Phenotype of the *GhCHLI* -silenced plants by VIGS. (A) The leaves of TM-1 turned yellow after *GhCHLI* gene silencing, and empty vector (*TRV:00*) leaves remained as green as the wild-type TM-1 when the leaves of *TRV1:CLA* had turned white. (B) The expression level of the *CHLI* gene in the *GhCHLI*-silenced leaves was much lower, as determined by real-time quantitative reverse transcription (qRT)-PCR analysis. Student’s *t*-test: **P<0.01. (C) Chlorophyll content was lower in the VIGS plants compared with TM-1 plants. Student’s *t*-test: **P<0.01.

To further investigate whether a mutation in *GhChlI* is responsible for the *v*_*1*_ phenotype, we cloned the 3′-end fragment (−284 bp) of *GhChlI_D10* from TM-1 and inserted it into the pTRV2 vector for VIGS so as to suppress its expression *in vivo* (Ma *et al.*, 2016). Fourteen days after agroinfiltration, the young leaves of the *GhChlI*-silenced TM-1 plants changed from green to yellow, and the young leaves of the TM-1 and TRV:00 (TM-1 infiltrated with empty vector) plants remained green (Fig. 5A). We performed two independent VIGS experiments. Of 60 VIGS plants, the young leaves of 56 plants turned yellow like the *v*_*1*_ mutant. qRT-PCR analysis showed that *GhChlI* was dominantly down-regulated in the leaves of the *GhChlI*-silenced plants as compared with the control (Fig. 5B). Furthermore, the levels of chlorophyll *a* and chlorophyll *b* were much lower in the *GhChlI*-silenced TM-1 plants compared with the controls (Fig. 5C). These results convincingly demonstrate that *GhChlI* is the gene responsible for the *v*_*1*_ phenotype.

## Discussion

### BSA-seq is an efficient strategy for rapid gene cloning

Map-based cloning is an important and effective strategy for gene cloning. However, the general strategy for map-based cloning is time-consuming and laborious due to the need to develop a large population comprising tens of thousands of individuals to pinpoint the genetic variants and screen out a large number of molecular markers to identify the polymorphic DNA marker linked to the traits of interest. For example, a mapping population consisting of 12 000 F_2_ plants was used to identify the *D53* gene encoding a protein that acted as a repressor of strigolactones in rice (Zhou *et al.*, 2013); an F_2_ population containing 2248 individuals was used for the fine mapping of the recessive dialytic gene, *dl*, in tomatoes (Chang *et al.*, 2016*a*), and a mapping population consisting of 9497 F_2_ plants was used to identify the *w* gene controlling the white immature fruit color in cucumbers (Liu *et al.*, 2016). But for some larger crop plants such as cotton, which require a large area for planting, growing so many progenies in the field can be impractical. In this study, we introduce an improved BSA-seq method that integrates the traditional BSA method with whole genomic resequencing and can be applied to rapidly identify specific genomic regions for a given trait from crops with assembled genomes, such as cotton. In the case of BSA-seq, it is only necessary to cross the mutant plant displaying the phenotype of interest with the wild-type plant in order to develop a mapping population, such as F_2_ or BC_1_. Then, a mutant-type DNA pool is constructed using the mutant type individuals of the population. The pool and the parent DNA were subjected to whole genome sequencing in order to produce several short reads. The sequences of the two parents are aligned to the reference genome so as to find SNPs between the two parents. Then, according to the difference ratio of the SNPs in the sequenced individuals, the genomic regions harboring the gene locus for the given traits are identified. In comparison, it has some advantages over other mapping methods. BSA-seq could solve the problem of limited polymorphisms between closer related parents. Based on whole genome sequencing, a large amount of SNPs and Indel markers were developed that ensure the number of nucleotide polymorphisms would be detected between the two materials. Thus, closer related parents could be used for developing mapping populations. Moreover, BSA-seq is fast, cheap and suitable for a variety of mapping populations. In our study, we spent only 6 months on the identification and functional verification of the candidate gene underlying the *v*_*1*_ locus using the integrated BSA-seq and VIGS strategy. With the completion of genome sequencing of more and more crops, the functional analysis of several unknown genes has become one of the most focused research areas. We anticipate that this method will expedite the map-based cloning of more agronomically important genes in crops.

### The functional role of *GhCHLI* in virescent trait in cotton

Leaf color mutants are often seen in higher plants. Leaf color-related genes have been isolated from many plants, including Arabidopsis (Kim *et al.*, 2009), rice (Deng *et al.*, 2012), and cucumber (Liu *et al.*, 2016). Most formerly cloned leaf color-related genes are involved in chloroplast development, or the synthesis or degradation of chlorophyll, and thus directly influence photosynthesis and plant growth and development. In cotton, 26 gene loci from 22 leaf-color mutants have been identified, and even some loci that interacted with each other (Zhang *et al.*, 1997). These mutants differ in the yellowness level of their leaves, indicating the diverse mechanisms of leaf-color mutations. Therefore, these mutants are suitable material for studying the mechanism of leaf color formation. Of the 26 gene loci, 11 have been mapped to linkage groups or chromosomes (Tian, 2011), but no items have been cloned. Virescent-1 was the first identified leaf-color mutant displaying a greenish yellow appearance at the seedling stage. This characteristic is indicative of intraspecific heterosis in upland cotton and can be used to distinguish pseudo-hybrid F_1_ generations, and thus simplify the process of hybrid seed production. In this study, we successfully identified the exact gene underlying the *v*_*1*_ locus as encoding the CHLI subunit of magnesium chelatase I. Mg chelatase I contains CHLI, CHLD and CHLH subunits in dicot plants, and it presents a catalytic activity if all the subunits are combined. Two of the three subunits, CHLI and CHLD, include a similar ATPase structure and an AAA+ domain in the N-terminus, but the CHLD shows no ATPase activity (Jensen *et al.*, 1999; Fodje *et al.*, 2001). Single amino acid mutations of the CHLI in the AAA+ domain in higher plants, such as Arabidopsis (Huang and Li, 2009), barley (Hansson *et al.*, 1999), maize (Sawers *et al.*, 2006), soybean (Campbell *et al.*, 2015), rice (Zhang *et al.*, 2006, 2015*a*), and cucumber (Gao *et al.* 2016) have been demonstrated to disrupt the activity of Mg-chelatase. In our study, we also found the SNP resulting in the amino acid residue change in the AAA+ domain of *GhCHLI* in T582. In addition, we also observed the reduction in the number of *GhCHLI* transcripts in T582 compared with TM-1. However, it remains to be explored whether the mutation in the coding region of *GhCHLI* leads to protein structure variation or the reduced transcript level causes the loss of *GhCHLI* function.

## Supplementary data

Supplementary data are available at *JXB* online.

Fig. S1. Multiple sequence alignment of the ORF1 genomic sequences from TM-1, T582 and coding sequence from TM-1.

Fig. S2. Multiple sequence alignment of the ORF2 genomic sequences from TM-1, T582 and coding sequence from TM-1.

Fig. S3. Multiple sequence alignment of the ORF3 genomic sequences from TM-1, T582 and coding sequence from TM-1.

Fig. S4. Multiple sequence alignment of the ORF4 genomic sequences from TM-1, T582 and coding sequence from TM-1.

Fig. S5. Multiple sequence alignment of the ORF5 genomic sequences from TM-1, T582 and coding sequence from TM-1.

Fig. S6. Multiple sequence alignment of the ORF6 genomic sequences from TM-1, T582 and coding sequence from TM-1.

Fig. S7. Multiple sequence alignment of the ORF7 genomic sequences from TM-1, T582 and coding sequence from TM-1.

Fig. S8. Multiple sequence alignment of the ORF8 genomic sequences from TM-1, T582 and coding sequence from TM-1.

Fig. S9. PCR products of ORF1 amplified from TM-1, T582, F1 and part virescent F2 individuals.

Fig. S10. PCR products of ORF4 amplified from TM-1, T582, F1 and part virescent F2 individuals.

Fig. S11. Sequence alignment of ORF4 genomic DNA from TM-1, T582 and 20 virescent F2 individuals.

Fig. S12. Sequence alignment of the *GhCHLI* promoter from TM-1 and T582.

Table S1. Primers used in *v*_*1*_ gene cloning in this study.

Table S2. Physical intervals that span the candidate genes for five mutant loci as identified by BSA-seq.

Table S3. Eight candidate ORFs and their putative functions.

## Author Contributions

TZ conceptualized and coordinated the project. JZ, FG, CX, HW, KC, and ZS conducted mapping, cloning and validation through VIGS of the *v*_*1*_ gene. JC conducted bioinformatics analysis of BSA-seq data. YH constructed DNA sequencing libraries and performed the genome sequencing. JZ, JC, YH, and TZ wrote the manuscript. All authors discussed results and commented on the manuscript.

## Conflict of interests

The authors declare no conflicts of interests.

## Supplementary Material

Supplementary_Figures_S1_S12_Tables_S1_S3

## Abbreviations:

BSA: bulked segregant analysis
*v*_*1*_: virescent-1
cu: cup leaf
*gl*_*1*_: glandless-1
fg: frego bract
*cl*_*1*_: cluster-1
BSA-seq: BSA and next generation sequencing
VIGS: virus-induced gene silencing.
