# Supplementary material for: Rapid mapping and cloning of the virescent-1 gene in cotton by bulked segregant analysis–next generation sequencing and virus-induced gene silencing strategies
Source: J Exp Bot. 2017 Jul 20;68(15):4125–35. doi: 10.1093/jxb/erx240 (PMC5853531; doi:10.1093/jxb/erx240)
Supplement: Supplementary_Figures_S1_S12_Tables_S1_S3 [file erx240_suppl_supplementary_figures_s1_s12_tables_s1_s3.pdf]

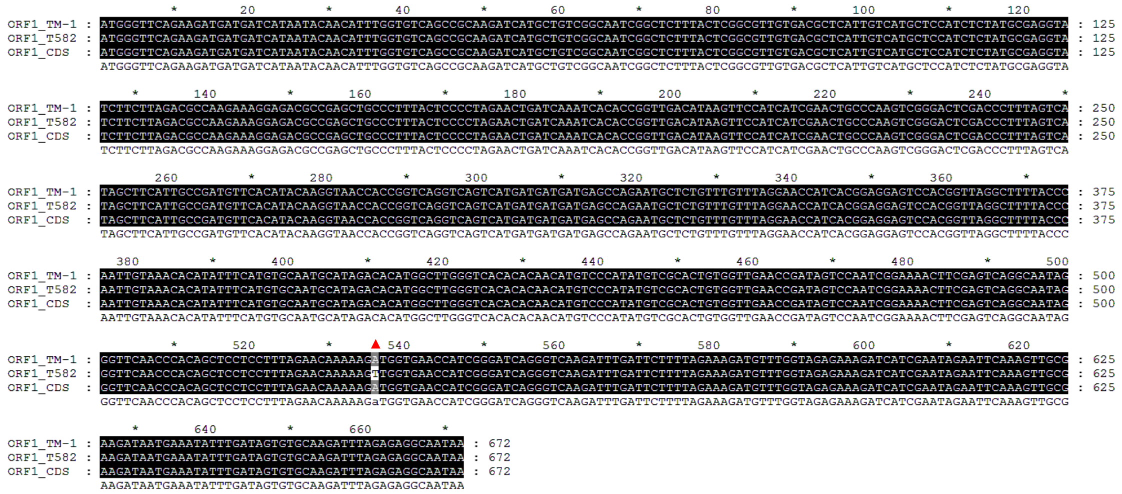

Fig. S1. Multiple sequence alignment of the ORF1 genomic sequences from TM-1, T582 and coding sequence from TM-1. The alignment was conducted using the Clustal X program. Black background indicates the exon. The red triangle shows the allelic variation in the exon between TM-1 and T582. The fragment was specifically amplified with primers D280 (**Table S1**).

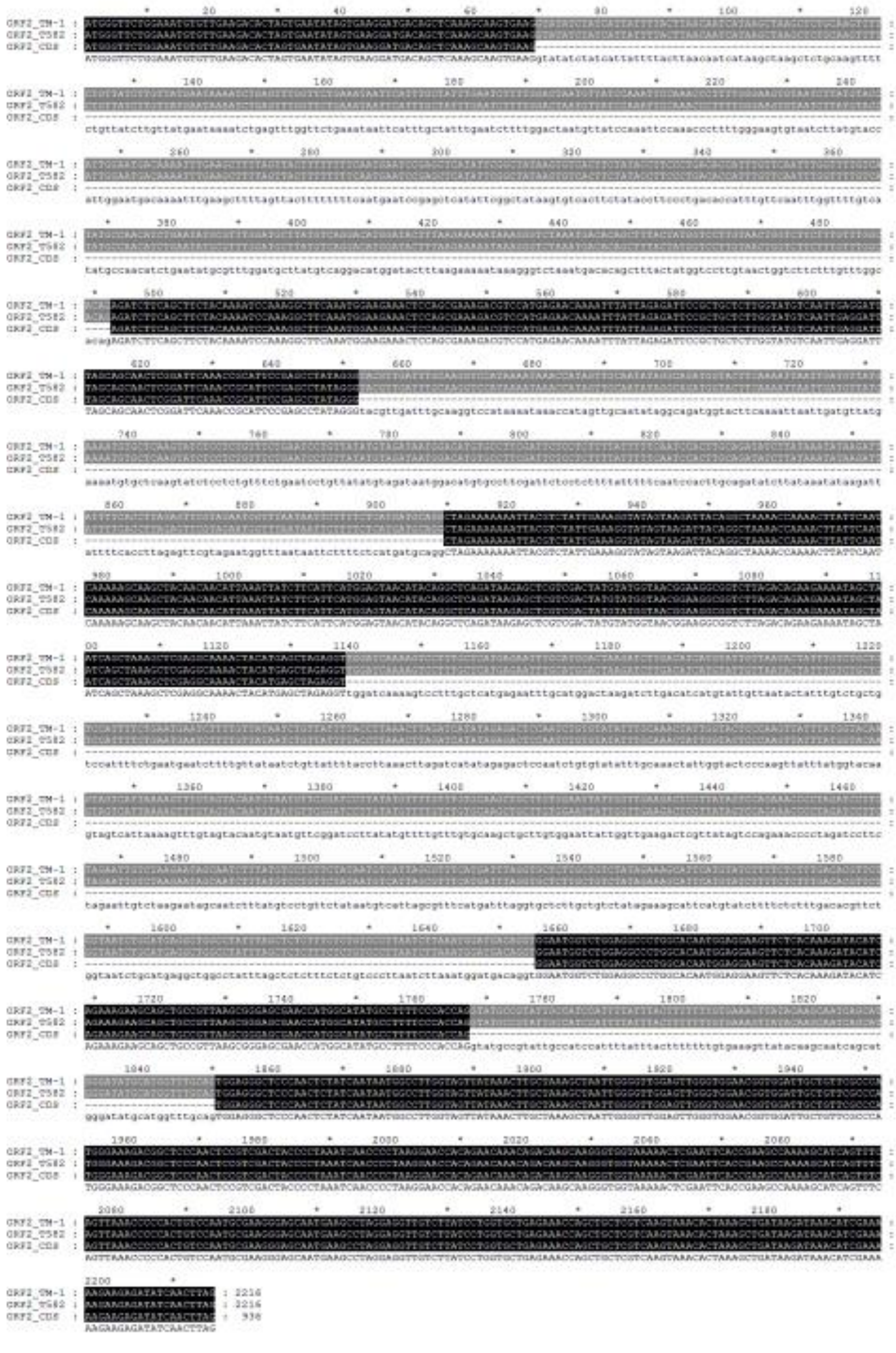

Fig. S2. Multiple sequence alignment of the ORF2 genomic sequences from TM-1, T582 and coding sequence from TM-1. The alignment was conducted using the Clustal X program. Black background indicates the exons; grey background indicates the introns. The fragment was specifically amplified with primers D281 (**Table S1**).

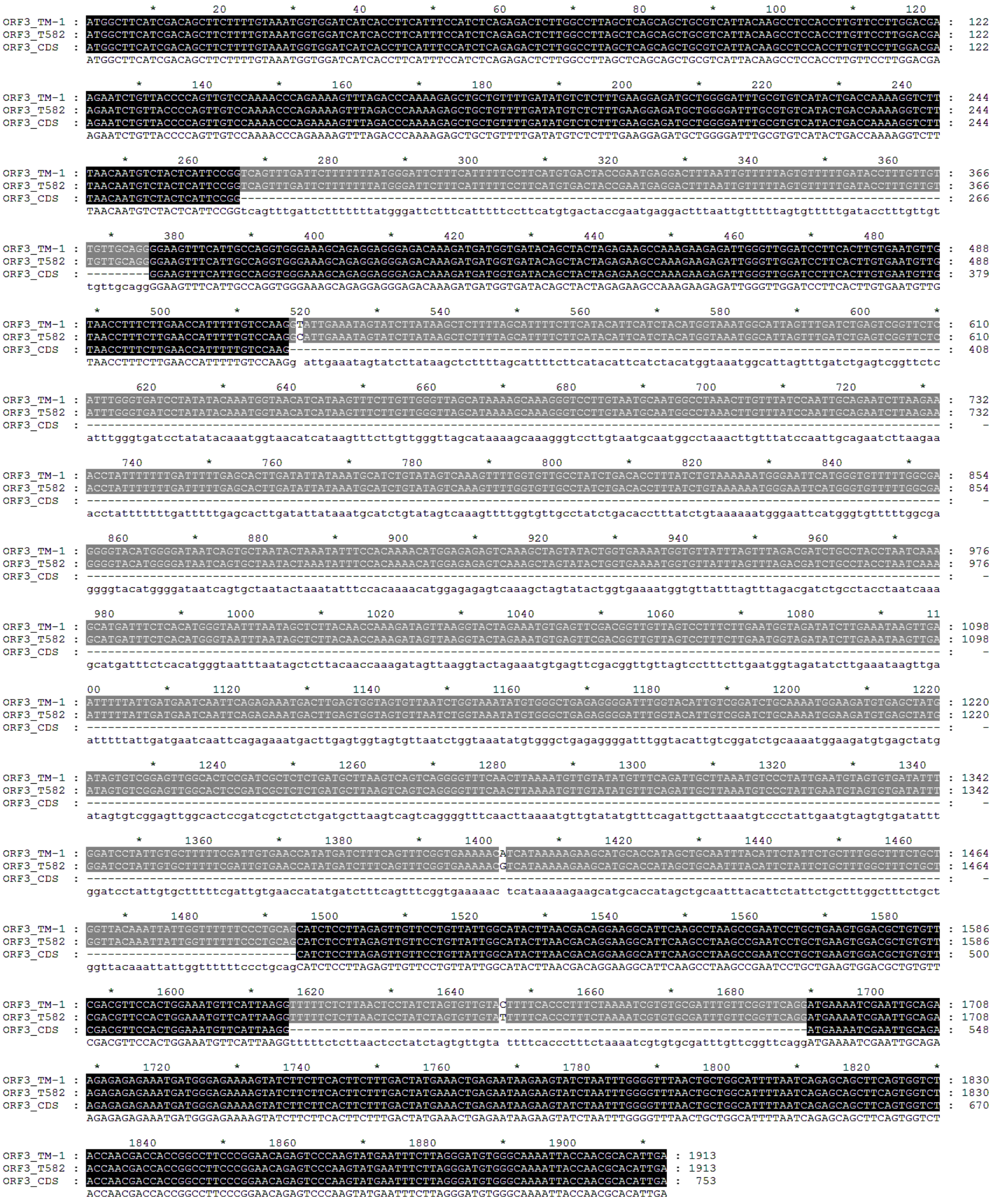

Fig. S3. Multiple sequence alignment of the ORF3 genomic sequences from TM-1, T582 and coding sequence from TM-1. The alignment was conducted using the Clustal X program. Black background indicates the exons; grey background indicates the introns. The fragment was specifically amplified with primers D282 (**Table S1**).

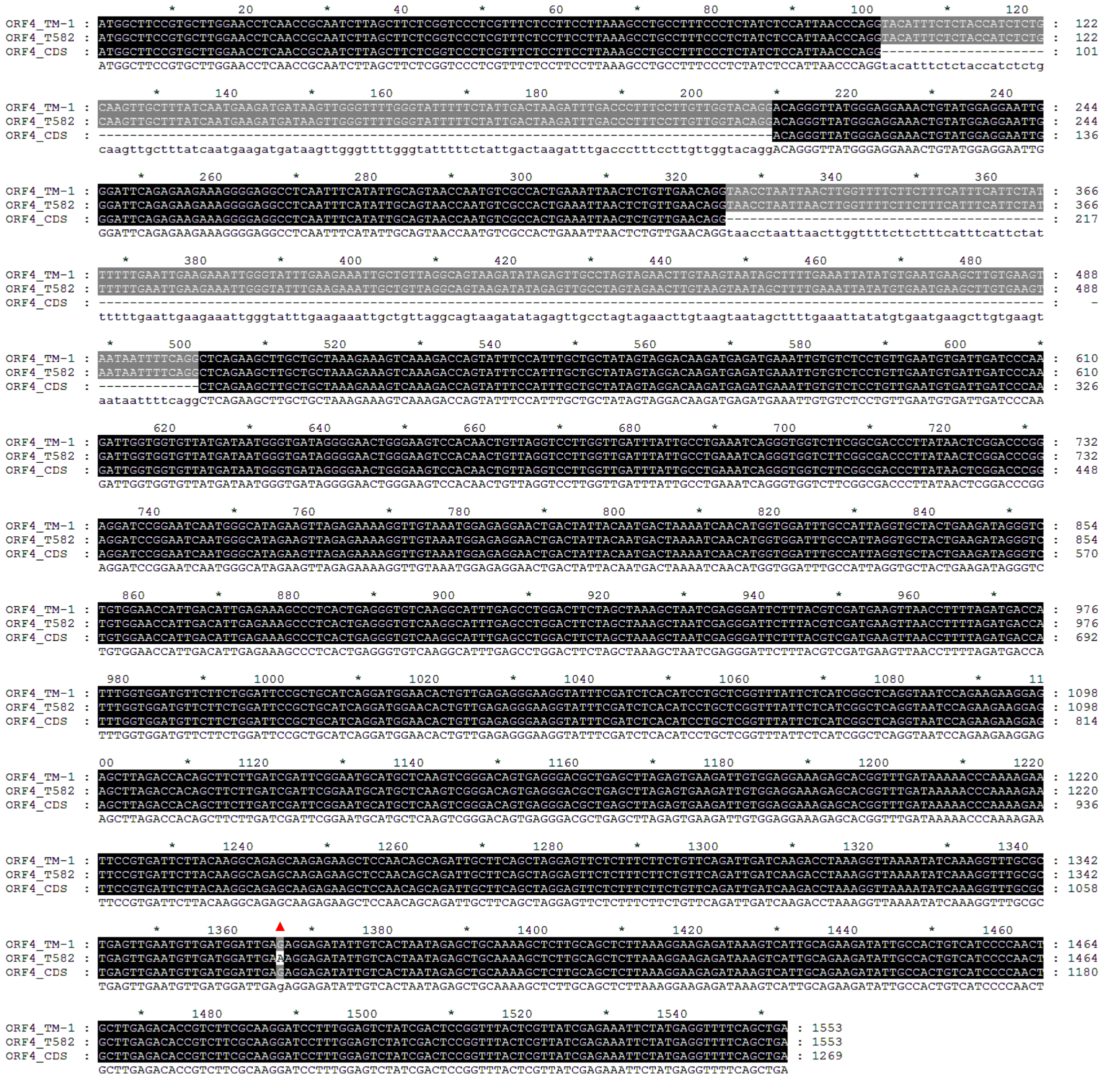

Fig. S4. Multiple sequence alignment of the ORF4 genomic sequences from TM-1, T582 and coding sequence from TM-1. The alignment was conducted using the Clustal X program. Black background indicates the exons; grey background indicates the introns. The red triangle shows the allelic variation between TM-1 and T582. The fragment was specifically amplified with primers K7772 (**Table S1**).

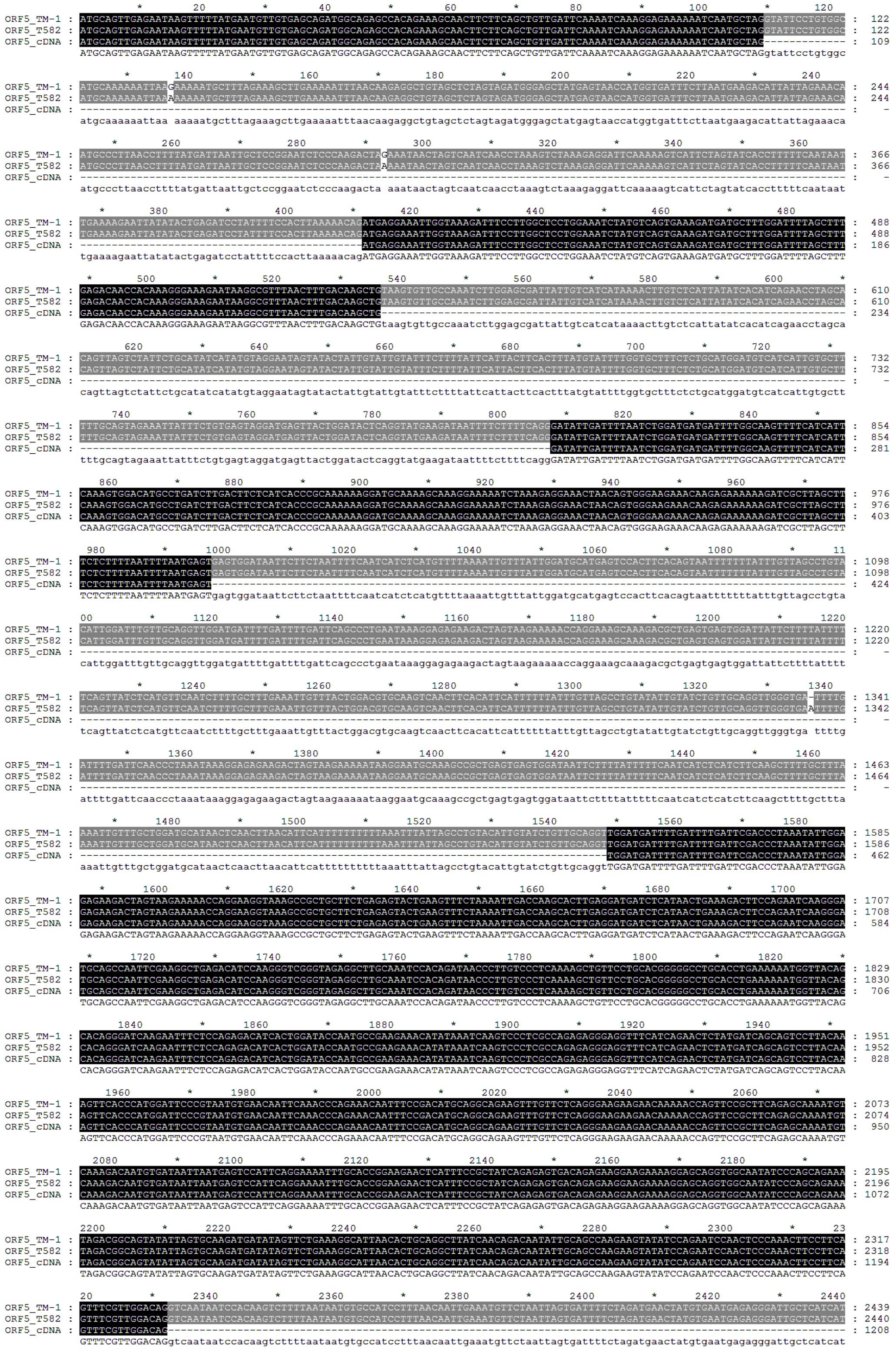
ORF5_TM-1
ORF5_T582
ORF5_cDNA

Continued

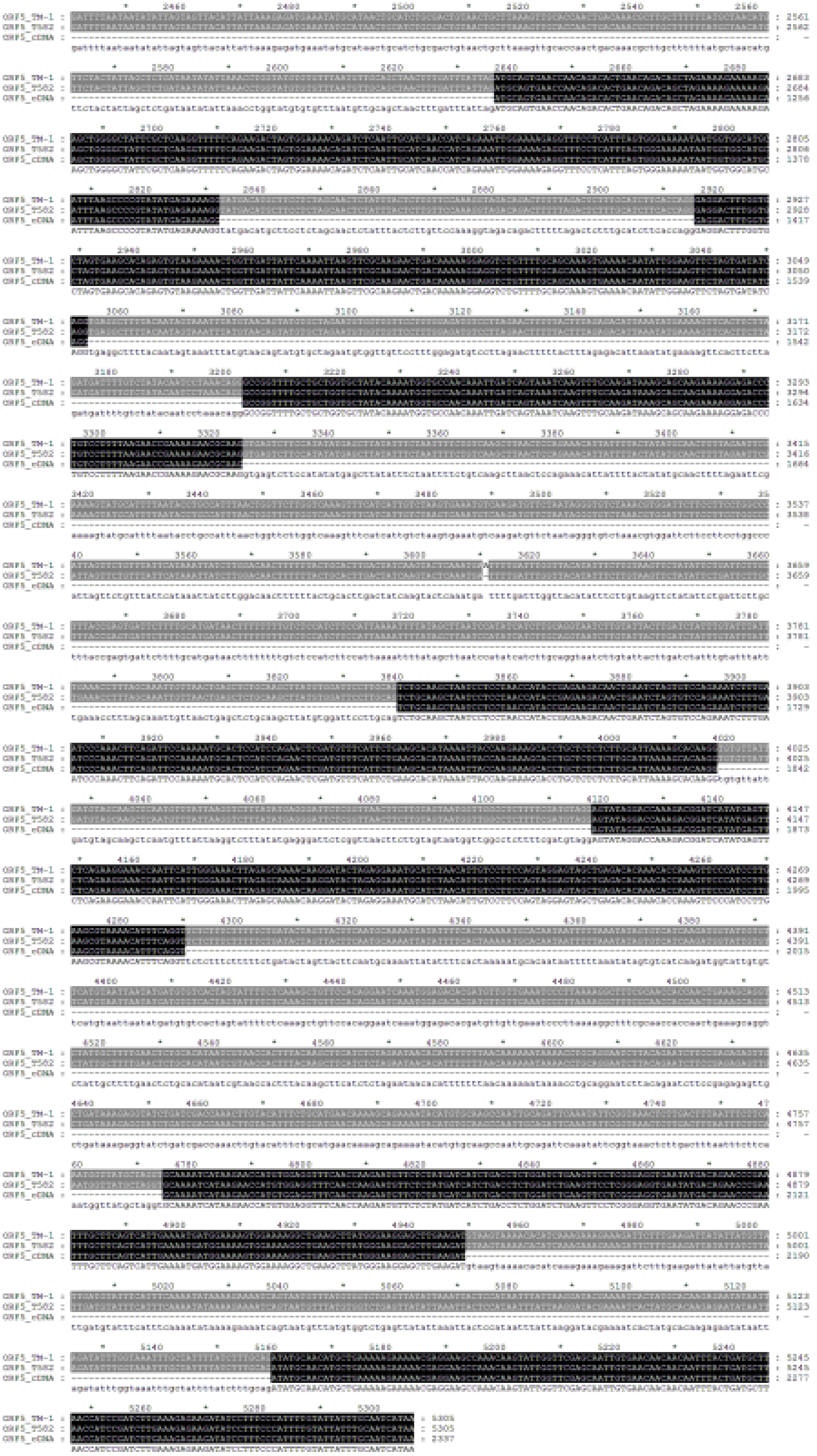

Fig. S5. Multiple sequence alignment of the ORF5 genomic sequences from TM-1, T582 and coding sequence from TM-1. The alignment was conducted using the Clustal X program. Black background indicates the exons; grey background indicates the introns. The fragment was specifically amplified with primers D284 (**Table S1**).

```
                    *        20         *        40         *        60         *        80         *       100         *       120
ORF6_TM-1 : ATGGCTATTCTCTCCTTCCATGAAAATGACAACAAGGAGAACATCCCTCCTTTCTCCTCAAAAAAGCCTACATTTCTTCTTACAAAACCTTCATCCTCCAACAATAAAAAAAGAAGATTAAGGAA : 125
ORF6_T582 : ATGGCTATTCTCTCCTTCCATGAAAATGACAACAAGGAGAACATCCCTCCTTTCTCCTCAAAAAAGCCTACATTTCTTCTTACAAAACCTTCATCCTCCAACAATAAAAAAAGAAGATTAAGGAA : 125
ORF6_CDS  : ATGGCTATTCTCTCCTTCCATGAAAATGACAACAAGGAGAACATCCCTCCTTTCTCCTCAAAAAAGCCTACATTTCTTCTTACAAAACCTTCATCCTCCAACAATAAAAAAAGAAGATTAAGGAA : 125
            ATGGCTATTCTCTCCTTCCATGAAAATGACAACAAGGAGAACATCCCTCCTTTCTCCTCAAAAAAGCCTACATTTCTTCTTACAAAACCTTCATCCTCCAACAATAAAAAAAGAAGATTAAGGAA

                 *       140         *       160         *       180         *       200         *       220         *       240         *
ORF6_TM-1 : ACCCCTTCAAGACATTACCAATCTTATTCTCCCACAAATCATCTCAACTCCAGTTCAATCCGATACAACAGTTCTGGTTTCTTCCCCAGCTTTGGCTTCTCAACCCAATTTCAAAAAGAGGAGAG : 250
ORF6_T582 : ACCCCTTCAAGACATTACCAATCTTATTCTCCCACAAATCATCTCAACTCCAGTTCAATCCGATACAACAGTTCTGGTTTCTTCCCCAGCTTTGGCTTCTCAACCCAATTTCAAAAAGAGGAGAG : 250
ORF6_CDS  : ACCCCTTCAAGACATTACCAATCTTATTCTCCCACAAATCATCTCAACTCCAGTTCAATCCGATACAACAGTTCTGGTTTCTTCCCCAGCTTTGGCTTCTCAACCCAATTTCAAAAAGAGGAGAG : 250
            ACCCCTTCAAGACATTACCAATCTTATTCTCCCACAAATCATCTCAACTCCAGTTCAATCCGATACAACAGTTCTGGTTTCTTCCCCAGCTTTGGCTTCTCAACCCAATTTCAAAAAGAGGAGAG

                   260         *       280         *       300         *
ORF6_TM-1 : ATGAAGATAAGCTAGGATCAATTTGCAGGAAGACTCATTTCGTGTATAAAAGTGTGAACTTCCGATAG : 318
ORF6_T582 : ATGAAGATAAGCTAGGATCAATTTGCAGGAAGACTCATTTCGTGTATAAAAGTGTGAACTTCCGATAG : 318
ORF6_CDS  : ATGAAGATAAGCTAGGATCAATTTGCAGGAAGACTCATTTCGTGTATAAAAGTGTGAACTTCCGATAG : 318
            ATGAAGATAAGCTAGGATCAATTTGCAGGAAGACTCATTTCGTGTATAAAAGTGTGAACTTCCGATAG
```

Fig. S6. Multiple sequence alignment of the ORF6 genomic sequences from TM-1, T582 and coding sequence from TM-1. The alignment was conducted using the Clustal X program. Black background indicates the exons. The fragment was specifically amplified with primers D285 (**Table S1**).

```
                 *        20         *        40         *        60         *        80         *       100         *       120
ORF7_TM-1 : ATGGGTAACTGGCGATTTCTTGCATGTTTGATACTGATTTTGTACTCATTTTCAAGCTCTGAATGTCGCCTTCTTAATCCAAACATTGACGGGAAAAACCCAACAGGCTCTTTCCGAATGTTGTC : 125
ORF7_T582 : ATGGGTAACTGGCGATTTCTTGCATGTTTGATACTGATTTTGTACTCATTTTCAAGCTCTGAATGTCGCCTTCTTAATCCAAACATTGACGGGAAAAACCCAACAGGCTCTTTCCGAATGTTGTC : 125
ORF7_CDS  : ATGGGTAACTGGCGATTTCTTGCATGTTTGATACTGATTTTGTACTCATTTTCAAGCTCTGAATGTCGCCTTCTTAATCCAAACATTGACGGGAAAAACCCAACAGGCTCTTTCCGAATGTTGTC : 125
            ATGGGTAACTGGCGATTTCTTGCATGTTTGATACTGATTTTGTACTCATTTTCAAGCTCTGAATGTCGCCTTCTTAATCCAAACATTGACGGGAAAAACCCAACAGGCTCTTTCCGAATGTTGTC

               *       140         *       160         *       180         *       200         *       220         *       240
ORF7_TM-1 : CCTTGCCACTAGTTCAGGTAAGGTGTATCAGTTTAATATAGGGATCGGAGATGAGTCTGGAAATGAGAACCTCTACGAATCAAAACGCCGAAGTCCAGGCGGTCCGGACCCGAAACATCACTGA : 249
ORF7_T582 : CCTTGCCACTAGTTCAGGTAAGGTGTATCAGTTTAATATAGGGATCGGAGATGAGTCTGGAAATGAGAACCTCTACGAATCAAAACGCCGAAGTCCAGGCGGTCCGGACCCGAAACATCACTGA : 249
ORF7_CDS  : CCTTGCCACTAGTTCAGGTAAGGTGTATCAGTTTAATATAGGGATCGGAGATGAGTCTGGAAATGAGAACCTCTACGAATCAAAACGCCGAAGTCCAGGCGGTCCGGACCCGAAACATCACTGA : 249
            CCTTGCCACTAGTTCAGGTAAGGTGTATCAGTTTAATATAGGGATCGGAGATGAGTCTGGAAATGAGAACCTCTACGAATCAAAACGCCGAAGTCCAGGCGGTCCGGACCCGAAACATCACTGA
```

Fig. S7. Multiple sequence alignment of the ORF7 genomic sequences from TM-1, T582 and coding sequence from TM-1. The alignment was conducted using the Clustal X program. Black background indicates the exons. The fragment was specifically amplified with primers D286 (**Table S1**).

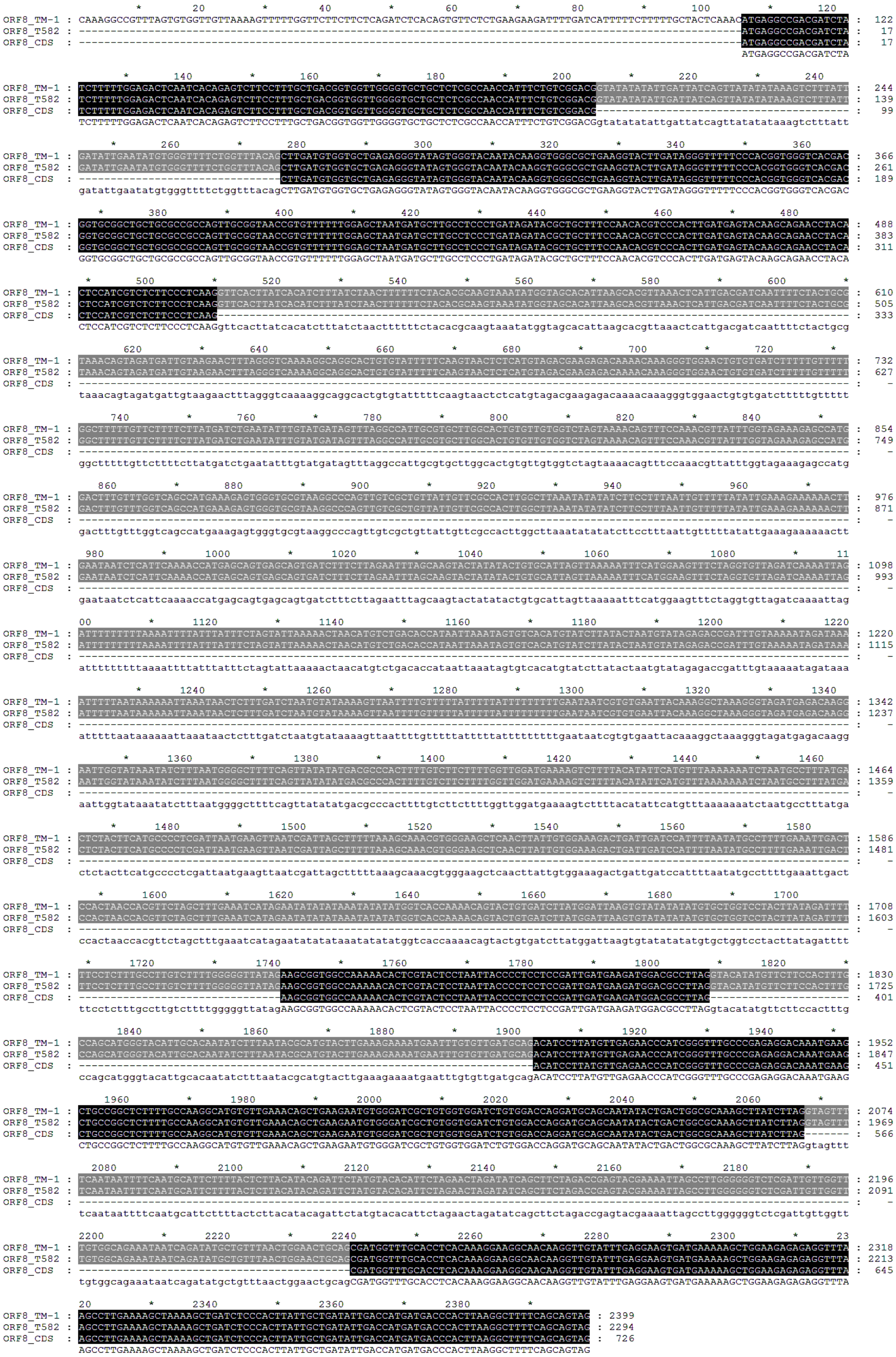

Fig. S8. Multiple sequence alignment of the ORF8 genomic sequences from TM-1, T582 and coding sequence from TM-1. The alignment was conducted using the Clustal X program. Black background indicates the exons; grey background indicates the introns. The fragment was specifically amplified with primers D287 (**Table S1**).

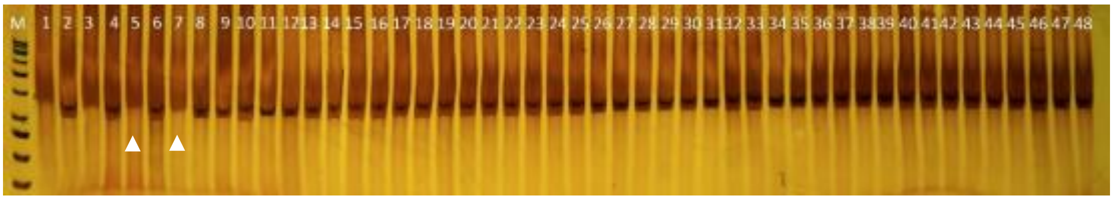

Fig. S9. PCR products of ORF1 amplified from TM-1, T582, F_1 and part virescent F_2 individuals. The numbers above each lane denote as following: 1, TM-1; 2, T582; 3, F_1 (TM-1×T582); 4-48, virescent F_2 individuals. Products were classified into T582-type and TM-1-type (no band). Because primers is specific for T582, only individuals containing the T582-type SNP can be amplified. M, 50bp ladder. The white triangle shows the two individuals containing the TM-1-type SNP but virescent trait. The fragment was specifically amplified with primers K9994 (**Table S1**).

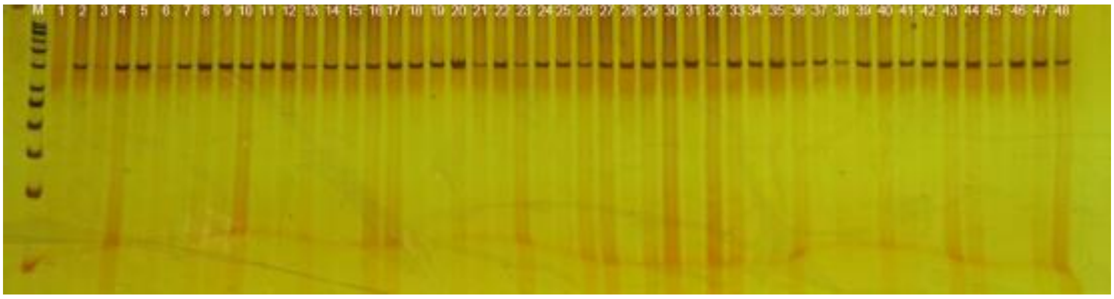

Fig. S10. PCR products of ORF4 amplified from TM-1, T582, F_1 and part virescent F_2 individuals. The numbers above each lane denote as following: 1, TM-1; 2, T582; 3, F_1 (TM-1×T582); 4-48, virescent F_2 individuals. Products were classified into T582-type and TM-1-type (no band). Because primers is specific for T582, only individuals containing the T582-type SNP can be amplified. M, 50bp ladder. The SNP was consistent in the mutant F_2 individuals. The fragment was specifically amplified with primers K9441 (**Table S1**).

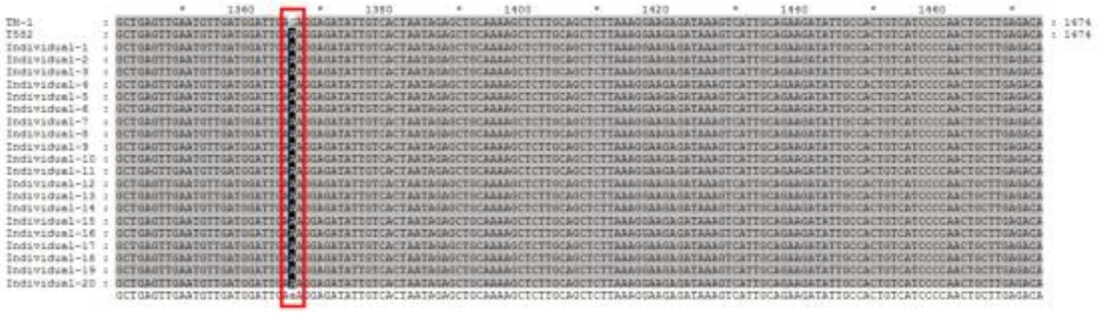

Fig.S11 Sequence alignment of ORF4 genomic DNA from TM-1, T582 and 20 virescent F_2 individuals. The fragment was specifically amplified with primers (K9993F 5’TCAGATTGATCAAGACCTAAAGGTTAAAA3’; K9993R 5’TGAAAACCTCATAGAATTTCTCGATAACG3’). The ORF4 primer pair flanked the fragment with the allelic variation (G in TM-1, A in T582) in the red box.

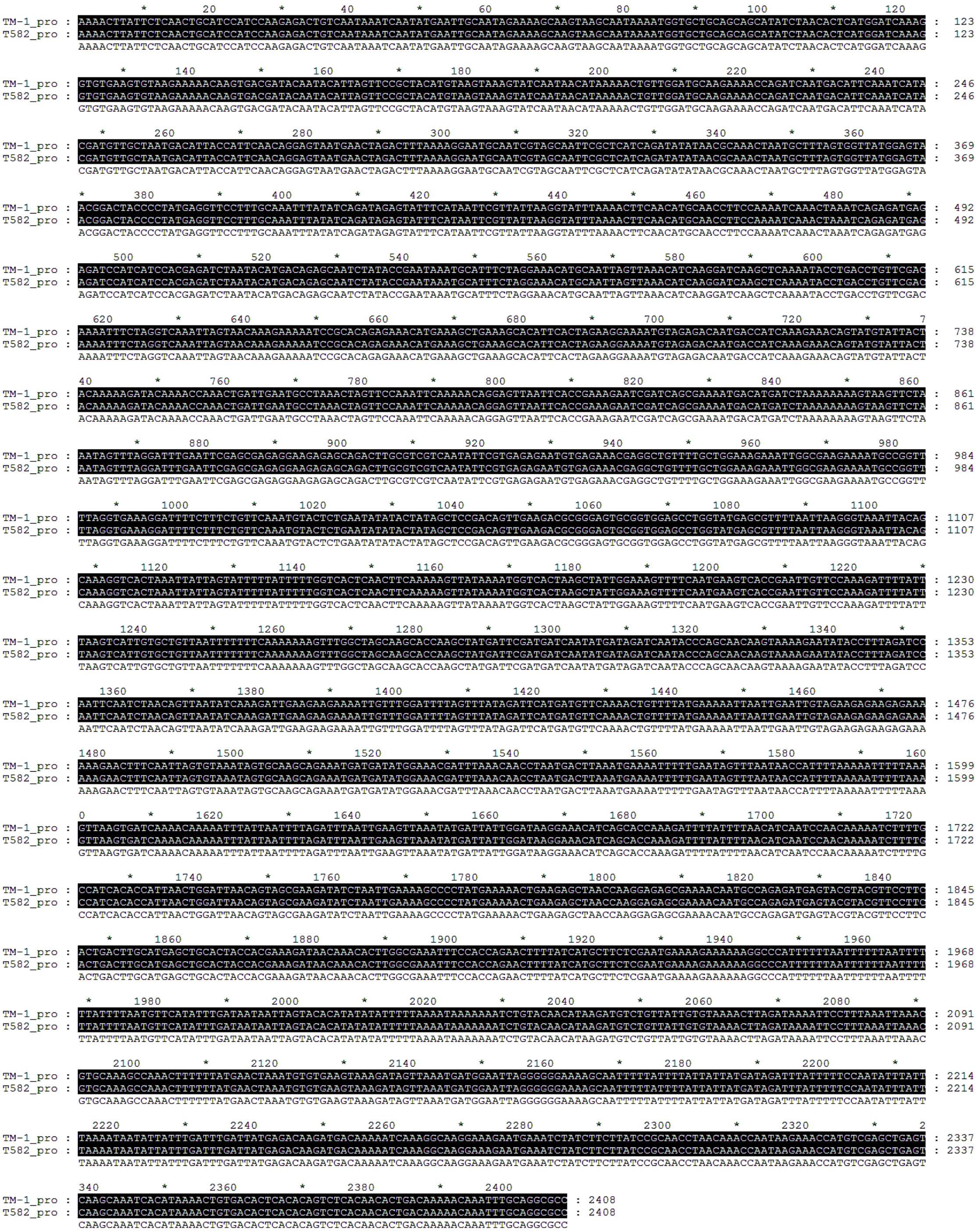

Fig.S12 Sequence alignment of the *GhCHLI* promoter from TM-1 and T582. The fragment was specifically amplified with primers (D283-promoter F: 5’GGATCTTCCAGAGATCAAGCACGGAAGCCATGGCGCCTGCAAATTTG3’; D283-promoter R: 5’CTGCCGTTCGACGATACAACATTCATAAAAACTTATTCTCAACTGCA3’).

Table S1. Primers used in *v_1* gene cloning in this study

Purpose	Primer names	Sequence(5'-3')
Indel primers for mapping virescent-1	K4815F	ACCTCATAGTGCCATGTCATCA
Indel primers for mapping virescent-1	K4815R	ACATTAGTGATTGTCGAGTACTGT
Indel primers for mapping virescent-1	K4818F	TTCGTGATGATGCCCGACAA
Indel primers for mapping virescent-1	K4818R	GCGGATTCTCTCATTCGGGA
Indel primers for mapping virescent-1	K4819F	AAAAAGCCTGGGTTTCTCGA
Indel primers for mapping virescent-1	K4819R	CGCTGCTCTTAGGAAGCAGT
Indel primers for mapping virescent-1	K4820F	TGGAGGTAAAGGCAAGTAGGG
Indel primers for mapping virescent-1	K4820R	TGGTAGGAGTAGGGTTTGGTTG
Indel primers for mapping virescent-1	K4821F	ACTTAGTGTTGCGGATAACTCT
Indel primers for mapping virescent-1	K4821R	GGCAGATGCTATGGCTGGAA
Indel primers for mapping virescent-1	K4822F	TTTGGACTGCCGAGGACTTT
Indel primers for mapping virescent-1	K4822R	TGGAAGTTCACGTCTGGCTC
Indel primers for mapping virescent-1	K4823F	GGCTGATCTCTCACAGGTCC
Indel primers for mapping virescent-1	K4823R	TGGATACATGGATCTGGTGCA
Indel primers for mapping virescent-1	K4824F	ACCGTGTATCAACAGCATCTGA
Indel primers for mapping virescent-1	K4824R	GCACCTGGGAATCGAATGGA
Indel primers for mapping virescent-1	K4825F	CAACTCACTGTCACCTACACCT
Indel primers for mapping virescent-1	K4825R	ACCTGAACTTTGGCATTGGC
Indel primers for mapping virescent-1	K4826F	CCATAATTCAAGCGGTGGCA
Indel primers for mapping virescent-1	K4826R	AGATCGAATTAAACTCATCCAACCA
Indel primers for mapping virescent-1	K5426F	TGCTTGACCATGGCAGTGAT
Indel primers for mapping virescent-1	K5426R	TGAAGCATAGTTTAGTGACATTGGA
Indel primers for mapping virescent-1	K5427F	AGCCTAGTAGGAATGCCCCT
Indel primers for mapping virescent-1	K5427R	CGGTTCTTCTCAAGATCTCCCC
Indel primers for mapping virescent-1	K5428F	TCATCTTTAAGGGGACTAAAGTGT
Indel primers for mapping virescent-1	K5428R	CCATGCCATTTCCCCCTTCT
Indel primers for mapping virescent-1	K5429F	TCCATGGCTAACGAGGTCCT
Indel primers for mapping virescent-1	K5429R	TCTCAACAGACGGACAGCTT
Indel primers for mapping virescent-1	K5430F	GAGAGGGTGTGATCTTTCTCCT
Indel primers for mapping virescent-1	K5430R	TCCCCCTTAATTTCTACAATAAGCT
Indel primers for mapping virescent-1	K5431F	CCGCAACTGGAGGATTAGCA
Indel primers for mapping virescent-1	K5431R	TCTAGCTCAGACGGCGTTTC
Indel primers for mapping virescent-1	K5432F	TGTGTATATACTTGTTTTCTTACCCCT
Indel primers for mapping virescent-1	K5432R	ACGTATAGTGTTTGAAGTACATATGAG
Indel primers for mapping virescent-1	K5433F	TGAGTGAAGAAATTAGTCCCGTGA
Indel primers for mapping virescent-1	K5433R	ACTCATCTTCCCGTGAAAGCA
Indel primers for mapping virescent-1	K5434F	AGGACCAGTCAGACTCAACTCT
Indel primers for mapping virescent-1	K5434R	CGTTGCACGTGGATCATTTGT
Indel primers for mapping virescent-1	K5435F	CGGGCAACTGCATGGAATTT
Indel primers for mapping virescent-1	K5435R	AGCCATTGGTGTCTTCCTGG
Indel primers for mapping virescent-1	K5482F	TGGGAGCACACACCAAAAGT

Indel primers for mapping virescent-1	K5482R	TTAGTGGCTGCAGTGCATGT
Indel primers for mapping virescent-1	K5483F	CGGTTCCAAGCCCTCACTAG
Indel primers for mapping virescent-1	K5483R	TTTCCTCTCGAAAGGGTTCAA
Indel primers for mapping virescent-1	K5484F	TCGTGATGTTTCCACGGACT
Indel primers for mapping virescent-1	K5484R	TCCAACTCTTAAACCGGAAACC
Indel primers for mapping virescent-1	K5485F	AGTTTGAGTGCGTTGAAGCA
Indel primers for mapping virescent-1	K5485R	ACGGTAAAAGTGATATGAGTGTCT
Indel primers for mapping virescent-1	K5486F	GAGGTGTTTATAGGTCGGGTCG
Indel primers for mapping virescent-1	K5486R	AAGCTCATATTTCGGGCCAGA
Indel primers for mapping virescent-1	K5487F	AGTGGGTTTGAGCTTGAGCA
Indel primers for mapping virescent-1	K5487R	TCCAATGTTAGCTCCGTGGT
Indel primers for mapping virescent-1	K5488F	CGGGTTCGAATTTTGTTGAGGA
Indel primers for mapping virescent-1	K5488R	TGATTAGGTTATGAGCCCCCG
Indel primers for mapping virescent-1	K5489F	TGCATTAATCCAGGATTTCACCT
Indel primers for mapping virescent-1	K5489R	TGATACTATGGTTGCTGCATAACA
Indel primers for mapping virescent-1	K5490F	TGTTACCGATTCGTGATTTTCTCT
Indel primers for mapping virescent-1	K5490R	TTTTAGGGGGAACATGGCCC
Indel primers for mapping virescent-1	K5491F	ATGTACTCTAGCTTGCGCCG
Indel primers for mapping virescent-1	K5491R	TGGGCAAAGCATAAGGAAAACG
Indel primers for mapping virescent-1	K5492F	TCCATGACAAGGGACACGAA
Indel primers for mapping virescent-1	K5492R	GAAGTTGAAGGGTCGAGGCA
Indel primers for mapping virescent-1	K5493F	GCAGATGCAACCCTGTCATTG
Indel primers for mapping virescent-1	K5493R	ACATTCCCTCCCGAAAGAAAAGA
Indel primers for mapping virescent-1	K5494F	ACATTAAAAGGCGGAGGGGC
Indel primers for mapping virescent-1	K5494R	TGGATTTGGGGAGGCTCAAC
Indel primers for mapping virescent-1	K5495F	TTCTGGGTTTGGATGGGCTG
Indel primers for mapping virescent-1	K5495R	TGCTCATTTGACAATCACGTAACA
Indel primers for mapping virescent-1	K5496F	GGTGACGGTCCATGCAAAAG
Indel primers for mapping virescent-1	K5496R	GACGTGCACTCTGGTGAGAA
Indel primers for mapping virescent-1	K5497F	TGATAAATTTGGCCACTAATGTTTGT
Indel primers for mapping virescent-1	K5497R	CGAGGCAAGTGTTCGGATCT
Indel primers for mapping virescent-1	K5498F	TCACTGTTATGTTGCTACTCTTTTGT
Indel primers for mapping virescent-1	K5498R	GATCCGGCTCGACCCATATT
Indel primers for mapping virescent-1	K5499F	AGCAAACCCATTTCCAAATCGT
Indel primers for mapping virescent-1	K5499R	CGGTAGGTCAACGAAGTAGCA
Indel primers for mapping virescent-1	K5501F	TTTTCACACTTCCGCTGTGG
Indel primers for mapping virescent-1	K5501R	AAAAACGCCACTAACGCTCG
Indel primers for mapping virescent-1	K5502F	CCACACCACCATGATCGACA
Indel primers for mapping virescent-1	K5502R	TGACATGGGGTTCAATGGGG
Indel primers for mapping virescent-1	K5504F	AGGTTGGCAGGTCGCTTAAA
Indel primers for mapping virescent-1	K5504R	GGGAGCCATAATGACAGGGG
Indel primers for mapping virescent-1	K5513F	TCCCCCTCTTCTTGTGTCCT
Indel primers for mapping virescent-1	K5513R	TTACCACAAGCTCGATCCGA
Indel primers for mapping virescent-1	K5514F	GTAACAGAGGACCTACGCGG

Indel primers for mapping virescent-1	K5514R	AGGAAAAGCCTCTGTGCTCA
Indel primers for mapping virescent-1	K5515F	GGTATTCCACCCTTCTCTCTTTCT
Indel primers for mapping virescent-1	K5515R	ACTGCTATTCCAATTTCCAAGTCG
Indel primers for mapping virescent-1	K5516F	TTCCGTGGAGGCATTAAGCT
Indel primers for mapping virescent-1	K5516R	GGAAGGTACGCATTTGAGTGC
Indel primers for mapping virescent-1	K5517F	TGCATTTGATCAGATTTGAGGACA
Indel primers for mapping virescent-1	K5517R	GTGGCTAAAATGAAAGCACCCT
Indel primers for mapping virescent-1	K5518F	GACTAGTGCAGTCCATTGCG
Indel primers for mapping virescent-1	K5518R	TCTTGTTGAAATAGCACCACTTGT
Indel primers for mapping virescent-1	K5519F	ACCTTCTAACTTAACATGCCATGA
Indel primers for mapping virescent-1	K5519R	GACCCTAACTCGTGTCAATTACT
Indel primers for mapping virescent-1	K5520F	CCACTCCCAGAACCAGAAGC
Indel primers for mapping virescent-1	K5520R	CGTCTCAAAGGTTGCATGGC
Indel primers for mapping virescent-1	K5521F	AATCTTTTCAACTCAAGTCACACTT
Indel primers for mapping virescent-1	K5521R	GCTTGGAGAAAAGGCAGTGG
Indel primers for mapping virescent-1	K5522F	AAAACCCCGAGTGGTACTGC
Indel primers for mapping virescent-1	K5522R	TGTTGTGCTTGTCATAGGGCT
Indel primers for mapping virescent-1	K5523F	TGTTTCCTAGTGTTGCCCCC
Indel primers for mapping virescent-1	K5523R	CCCCTGTTAACCCTTTGGCT
Indel primers for mapping virescent-1	K5843F	TCCCTTGTTATCCCACGAAACA
Indel primers for mapping virescent-1	K5843R	TCGTTACCATGTTGGGTTTTCT
Indel primers for mapping virescent-1	K5844F	GCGCTCTAACTCCCCTCAAA
Indel primers for mapping virescent-1	K5844R	GCAGGGTGAAGTCAGAAGCT
Indel primers for mapping virescent-1	K5845F	GGGCTAATATGTCATGCCAGC
Indel primers for mapping virescent-1	K5845R	AGGGCAAGCAACCAATATCCA
Indel primers for mapping virescent-1	K5846F	AGGACCAGTCAGACTCAACTCT
Indel primers for mapping virescent-1	K5846R	CGTTGCACGTGGATCATTTGT
Indel primers for mapping virescent-1	K5847F	ATTATGGGTGCTGGCCTGTC
Indel primers for mapping virescent-1	K5847R	TGACAGGCTAGCACCCAAAT
Indel primers for mapping virescent-1	K5848F	GTTGATCCAAAGAGACGCGC
Indel primers for mapping virescent-1	K5848R	CGGATCGTGTCGGATGTTGA
Indel primers for mapping virescent-1	K5849F	CCGGCACCCAATCTGATTCT
Indel primers for mapping virescent-1	K5849R	TGGGTACTGGCCTGTCAATG
Indel primers for mapping virescent-1	K5850F	CCATTGATAGGCCAGCACCA
Indel primers for mapping virescent-1	K5850R	TGGGTACTGGCCTGTCAATG
Indel primers for mapping virescent-1	K5851F	CAGGCCAGCACCCAAAAATT
Indel primers for mapping virescent-1	K5851R	GGCCGACACCCAACAGTAAA
Indel primers for mapping virescent-1	K5852F	GGTGTAGTCGCAACTCTTTTTGT
Indel primers for mapping virescent-1	K5852R	CTTTTCGCTAGGGCTGGGAA
Indel primers for mapping virescent-1	K6170F	GGGGTGTGTGGTTTGGAGAT
Indel primers for mapping virescent-1	K6170R	CCAACAATTGAAGGCCGCAA
Indel primers for mapping virescent-1	K6171F	CGAGTCAGGAACCAACAGCT
Indel primers for mapping virescent-1	K6171R	GGGTCCCCACTTTTCCGAAA
Indel primers for mapping virescent-1	K6172F	TCGGTACATTATGGCTTTTTAGTGA

Indel primers for mapping virescent-1	K6172R	AGGAGGAAAGGGTAGAGGGT
Indel primers for mapping virescent-1	K6173F	AGATCCGAACACTTGCCTCG
Indel primers for mapping virescent-1	K6173R	GCCCAACCTAGCCCATTCTT
Indel primers for mapping virescent-1	K6174F	TGCTAACATGTATACACCGGTCT
Indel primers for mapping virescent-1	K6174R	TGCAAATTCAGGCTAGGTCA
Indel primers for mapping virescent-1	K6175F	CGAGCCCGAACCTATCAATCA
Indel primers for mapping virescent-1	K6175R	CGGCCCATGAGCACCTTTAT
Indel primers for mapping virescent-1	K6176F	CTCTGGGATCCACTGTCAGC
Indel primers for mapping virescent-1	K6176R	ACCGTCTAATTATTTCTTCAACTGCA
Indel primers for mapping virescent-1	K6177F	AGATTGTAAGGTATGGCAGCA
Indel primers for mapping virescent-1	K6177R	GCCGGTAAGTTTCATGTGCC
Indel primers for mapping virescent-1	K6178F	GTGCTGGGTGTTAGTTGGGT
Indel primers for mapping virescent-1	K6178R	TGGTGTCACTCATATACACAGTT
Indel primers for mapping virescent-1	K6179F	TGCGCAGATACACCTGAACT
Indel primers for mapping virescent-1	K6179R	ACACGCAGACTTTCATGTTAACA
Indel primers for mapping virescent-1	K6180F	ACTTTTCCATCCAAAAACATGCA
Indel primers for mapping virescent-1	K6180R	TGGCCCTATACTTTCGCTGT
Indel primers for mapping virescent-1	K6181F	TGGTACCACCATTTACAATGAAAA
Indel primers for mapping virescent-1	K6181R	TGGAGACCGCGTTAATGATT
Indel primers for mapping virescent-1	K6182F	ATTCTCTCGGCCTCTCAGGT
Indel primers for mapping virescent-1	K6182R	AAGCGTCAGTCATCTGGTGG
Indel primers for mapping virescent-1	K6183F	ACTTTGTTGGATTGCTTTCACTGT
Indel primers for mapping virescent-1	K6183R	TTTGATCCCCACAATGGCAC
Indel primers for mapping virescent-1	K6184F	AAGAAAGGCCAACGAGAGGG
Indel primers for mapping virescent-1	K6184R	CCTTACTTGCGGCTACTGCT
Indel primers for mapping virescent-1	K6185F	GCAACCAGCTGTGTGTAAGT
Indel primers for mapping virescent-1	K6185R	CAGTCAGCACTTGTGCCAAC
Indel primers for mapping virescent-1	K6186F	TGACTGATTGTTGCTGGAGCT
Indel primers for mapping virescent-1	K6186R	GCAAGCAGGAAAGCAAGAGG
Indel primers for mapping virescent-1	K6187F	TCAGCCAAACAAACAGACCCT
Indel primers for mapping virescent-1	K6187R	TCTCAGAGTGTCACGTGAACA
Indel primers for mapping virescent-1	K6188F	AGGAAAGTCCGCCCATGTTT
Indel primers for mapping virescent-1	K6188R	ACAAAACCCCAAACCCACCT
Indel primers for mapping virescent-1	K6189F	AGGCTGGCACACTTAATGAA
Indel primers for mapping virescent-1	K6189R	CCCAAAATTTTATGATAATTGGGCGA
Indel primers for mapping virescent-1	K6190F	TGGCCACTCAAATTTTCACATT
Indel primers for mapping virescent-1	K6190R	CTCTCCTAACCCATGTGACCA
SSR primers for mapping virescent-1	NAU2540F	AGCCACCCTGGAGAATATAA
SSR primers for mapping virescent-1	NAU2540R	TCCCCATTCATTTTGTAAAC
SSR primers for mapping virescent-1	CGR5565F	GCCATTAACCCATTAGGCAA
SSR primers for mapping virescent-1	CGR5565R	GCCATTGGAGCTTATAAGGATG
SNP primers for mapping virescent-1	K9441F	CGCTGAGTTGAATGTTGATGGATTGTA
SNP primers for mapping virescent-1	K9441R	AGAATCTACTTTGTGCTGAATATCTCACAATGTTT

SNP primers for mapping virescent-1	K9994F	GACCCTGATCCCGATGGTTCACCTA
SNP primers for mapping virescent-1	K9994R	CCCAAGTCGGGACTCGACCCTTTA
primers for Virus-induced gene silencing assay (VIGS)	K7002F	gtgagtaaggttaccgaattcCACTGTCCCGACTTGAGCAT
primers for Virus-induced gene silencing assay (VIGS)	K7002R	cgtgagctcggtaccggatccGGTGGATTTGCCATTAGGTG
primers for qRT-PCR ORF1	K7805F	GCCAGAATGCTCTGTTTGTTT
primers for qRT-PCR ORF1	K7805R	TGGACTATCGGTTCAACCACA
primers for qRT-PCR ORF2	K7393F	GGTAACGGAAGGCGGTCTTAG
primers for qRT-PCR ORF2	K7393R	GAGAACTTCCTCCATTGTGCC
primers for qRT-PCR ORF3	K7394F	TGCGTCATTACAAGCCTCCAC
primers for qRT-PCR ORF3	K7394R	TCAGTATGACACGCAAATCCC
primers for qRT-PCR ORF4	K7247F	AGGAAAGAGCACGGTTTGATA
primers for qRT-PCR ORF4	K7247R	CTCAGCGCAAACCTTTGATAT
primers for qRT-PCR ORF5	K7397F	ACTGGATACCAATGCCGAAGA
primers for qRT-PCR ORF5	K7397R	TTACGGGAATCCAAGGGTGAA
primers for qRT-PCR ORF6	K7399F	AACATCCCTCCTTTCTCCTCA
primers for qRT-PCR ORF6	K7399R	TGTTGTATCGGATTGAACTGG
primers for qRT-PCR ORF7	K7400F	TGTCGCCTTCTTAATCCAAAC
primers for qRT-PCR ORF7	K7400R	CCAGACTCATCTCCGATCCCT
primers for qRT-PCR ORF8	K7403F	TGGGTACAATACAAGGTGGGC
primers for qRT-PCR ORF8	K7403R	CAGCGTATCTATCAGGGAGGC
primers for test vector TRV1	TRV1F	TTGACTGATGTGCTGGGTTT
primers for test vector TRV1	TRV1R	CTTGGCTTTTACCTCGGATA
primers for test vector TRV2	TRV2F	TTGTTACTCAAGGAAGCACGAT
primers for test vector TRV2	TRV2R	TCCCCTATGGTAAGACAATGAG
primers for qRT-PCR(detect the quality of cDNA)	Y347F	AGACCACCAAGTACTACTGCAC
primers for qRT-PCR(detect the quality of cDNA)	Y347R	CCACCAATCTTGTACACATCC
primers for qRT-PCR(as an internal standard)	Y8991F	CGGTGGTGTGAAGAAGCCTCAT
primers for qRT-PCR(as an internal standard)	Y8991R	AATTTCACGAACAAGCCTCTGGAA
primers for cloning gene CHLI(ORF4)	K7802F	TCTCACAACACTGACAAAAACAAATTTGCAGGCGCC
primers for cloning gene CHLI(ORF4)	K7802R	ATCATGTTTGGAAAAAGGACTTCAGTAAGCATCTC
primers for cloning ORF1	D280F	ATGGGTTCAGAAGATGATGATCATAAT
primers for cloning ORF1	D280R	CCCTAAAGAAACATTAATACTAGGTG
primers for cloning ORF2	D281F	CCATGTCCATAGCTTAAGAAACTGA
primers for cloning ORF2	D281R	GGAGACATGGAATTGCAGTTGTCTGC
primers for cloning ORF3	D282F	CCAATCATTAATCTCATGGCTTCATCG
primers for cloning ORF3	D282R	GAAGTCCTTTTTCCAAACATGATTAGCT

primers for cloning ORF5	D284F	ggatcttccagagatGATTTATTGACAGTCTCTTGGATGGATGC
primers for cloning ORF5	D284R	ctgccgttcgacgatTAACTCCGACTAACTTTTGATGGAAG
primers for cloning ORF6	D285F	CTCAGTCAAATGGCTATTCTCTCCTTC
primers for cloning ORF6	D285R	GTGTTTGAGCTATCGGAAGTTCACAC
primers for cloning ORF7	D286F	CAAGTTAGAAGAAAATGGGTAACTGGCG
primers for cloning ORF7	D286R	GATGCCGATAACTACATATAGCTCTCAA
primers for cloning ORF8	D287F	ggatcttccagagatCAAAGGCCGTTTAGTGTGGTTGTTAAAAG
primers for cloning ORF8	D287R	ctgccgttcgacgatGTGGGTCATCATGGTCAATATCAGCAAT
primers for cloning promoter of ORF4	D283-promoterF	ggatcttccagagatCAAGCACGGAAGCCATGGCGCCTGCAAATTTG
primers for cloning promoter of ORF4	D283-promoterR	ctgccgttcgacgatACAACATTCATAAAAACTTATTCTCAACTGCA

Table S2. Candidate region for five mutant loci identified by BSA-seq

Character	Candidate region				Key region 1				Key region 2			
	Chr.	Start (Mb)	End (Mb)	Length (Mb)	Chr.	Start (Mb)	End (Mb)	Length (Mb)	Chr.	Start (Mb)	End (Mb)	Length (Mb)
virescent(v_1)	D10	0.70	3.90	3.20	D10	2.42	2.70	0.29	D10	2.87	3.37	0.51
cup leaf(cu)	A11	87.20	89.80	2.60	A11	88.46	88.48	0.02	A11	88.09	88.12	0.02
cluster boll(cl1)	D07	10.70	21.70	11.00	D07	14.42	14.65	0.24	D07	15.0	18.4	3.4
frego bract(fg)	A03	0.00	4.50	4.50	A03	0.31	3.47	3.16	A03	3.5	3.6	0.2
glandless (gl1)	D08	49.53	59.90	9.47	D08	53.75	55.80	2.05				

Table S3. Eight candidate ORFs and their putative functions

Candidate ORFs	ID	Start	End	Sense or antisense	Arabidopsis ID	Functional annotation
ORF1	Gh_D10G0280	2416685	2417356	-	AT2G42360	RING/U-box superfamily protein
ORF2	Gh_D10G0281	2428393	2430608	+	AT2G33990	IQ-domain 9
ORF3	Gh_D10G0282	2431254	2433166	+	AT1G28960	nudix hydrolase homolog 15
ORF4	Gh_D10G0283	2433422	2434360	+	AT5G45930	magnesium chelatase i2
ORF5	Gh_D10G0284	2436748	2442052	+	AT4G18490	Function unknown
ORF6	Gh_D10G0285	2443094	2443411	+		Function unknown
ORF7	Gh_D10G0286	2446306	2446554	+	AT1G73165	CLAVATA3/ESR-RELATE
ORF8	Gh_D10G0287	2451185	2453478	-	AT5G45920	SGNH hydrolase-type esterase superfamily protein
